# Research on the development strategy selection of the new energy vehicle industry from the perspective of green credit—Based on the foursquare evolutionary game analysis

**DOI:** 10.1371/journal.pone.0297813

**Published:** 2024-01-29

**Authors:** Jinlong Wang, Xiangbin Liu

**Affiliations:** 1 School of finance, Harbin University of Commerce, Harbin, Heilongjiang, China; 2 Accounting Department, Harbin Finance University, Harbin, Heilongjiang, China; National Textile University, PAKISTAN

## Abstract

Developing new energy vehicles is vital to promote green development and the harmonious coexistence of humans and nature. It is also the only way to help China move from a significant automobile country to a powerful automobile country. Based on the background of the "recession" of government subsidies and considering the importance of green credit in promoting green and low-carbon transformation, this paper constructs a four-party evolutionary game model that includes government, automotive companies, banks, and consumers to analyze the stability of the strategic choices of various parties in the development process of the new energy vehicle industry. It uses MATLAB simulation tools to analyze the impact of relevant factors on system stability. The research shows that: (1) The government’s subsidy mechanism significantly promotes the development of the new energy vehicle industry. Still, there is a subsidy threshold, beyond which the effect will weaken and quickly bring financial pressure. (2) With the gradual decline of government subsidies, the bank’s green credit policy has a specific policy complementary effect on the decline of government subsidies. (3) Considering that costs and benefits are the main influencing factors for automotive companies and consumers’ strategic choices, the impact of factors such as the punishment of violations, adjustment of subsidy policies, and consumers’ environmental awareness must also be paid attention to.

## 1. Introduction

Accelerating the development of the new energy vehicle industry is essential to deeply promote the energy revolution and accelerate the planning and construction of a new energy system. In 2020, the General Office of the State Council issued the "New Energy Vehicle Industry Development Plan (2021–2035)", which pointed out that the development of new energy vehicles is the only way for China to move from a significant automobile country to a powerful automobile country, and is a strategic measure to address climate change and promote green development. In July 2021, the Political Bureau of the Central Committee of the Communist Party of China proposed to "tap the potential of the domestic market and support the accelerated development of new energy vehicles", which shows that the development of new energy vehicles is crucial to promoting high-quality development of China’s economy. Since the "Plan for the Adjustment and Revitalization of the Automotive Industry" in March 2009, China has officially elevated the new energy automotive industry to an important strategic position for national development. The central government has provided subsidies through overall financial arrangements. The cumulative amount of subsidies exceeded 152.1 billion from 2010 to 2020. However, relying on national industrial policy support is not a long-term solution, especially with the emergence of "subsidy dependency" and "subsidy fraud" [1]. Since 2016, government subsidies have declined year by year. Given the importance of government subsidies for developing the new energy vehicle industry, the decline of subsidies will inevitably have a considerable impact on the new energy vehicle market [2]. According to data from the China Automobile Industry Association, since 2016, the sales growth rate of new energy vehicles has plummeted and has been further reduced. Even if the situation has eased in the later period, there are still some difficulties in comparing the growth rate brought about by the initial subsidy. In particular, in 2019, the production and sales of new energy vehicles decreased by 2.3% and 4% year-on-year. From January to October 2020, the production and sales of new energy vehicles decreased by 9.2% and 7.1% year-on-year, respectively, significantly narrowing by 9.5 and 10.6 percentage points compared to January to September, and the downward trend did not slow until November of that year.

In addition, as an essential financial link between the government and enterprises, green credit is of great significance for promoting the development of the green economy. Against the backdrop of declining subsidies year by year, according to the 2022 annual reports of car companies such as Ideal, Xiaopeng, and Weilai, it is still difficult for most car companies to rely solely on new energy vehicles to make money. However, due to continuously increasing research and development expenditures, infrastructure layout, and other factors, the financial constraints faced by car companies under dual pressure will become increasingly severe. At this time, green credit committed to supporting a green, low-carbon, and circular economy needs to fully play its complementary role in subsidizing the recession, which is crucial to alleviate the financing constraints of automotive enterprises, such as the Agricultural Bank of China’s new energy vehicle credit point usufruct pledge loan policy (briefly referred to as "green car loans"). The sustainable development-related loans of Pudong Development Bank to support the construction of charging infrastructure and the comprehensive financial services of Everbright Bank to assist the rapid development of new energy battery enterprises have been launched in succession, which shows the critical role of green credit policies in promoting the development of the new energy vehicle industry. Therefore, it is necessary to deeply study the issue of green credit to alleviate the financial constraints of the new energy vehicle industry. To fully play the complementary role of green credit in the decline of financial subsidies, it is of great practical significance to include banks as game players in the study of the stability of the strategic choices of stakeholders in the development of the new energy vehicle industry from the perspective of green credit.

The research of domestic and foreign scholars on the development of the new energy vehicle industry mainly focuses on policy promotion, influencing factors, etc. Regarding policy promotion, Chakraborty [3] and Jiao [4] conducted research from the perspective of "subsidized" policy tools, and Lin [5] studied the impact of "non-subsidy" policies on the promotion of new energy vehicles. Sun [6] and Jiang [7] considered the advancement and diffusion of new energy vehicles from the consumer demand perspective. As influencing factors, government subsidies are crucial to developing new energy vehicles [8–11]. In addition, the dual credit policy [12–14], industrial progress and external factors [15], production costs [16], technical maturity, and R&D funds [17] will have a significant impact on the development of the new energy vehicle industry. Therefore, it is necessary to conduct in-depth research on issues related to the further development of the new energy vehicle industry. In addition, with the rapid development of the economy, the development mode with oil and electricity as the primary energy structure has brought increasingly severe energy crises and environmental pollution problems [18]. Among them, as one of the main incentives, automobile fuel consumption, and exhaust emissions have received significant attention from countries worldwide and have also accelerated the development of the new global energy vehicle industry to a certain extent. The current global warming situation is still severe, and different scholars have conducted in-depth research from other countries, regions [19–23], and industries [24, 25] on reducing the excessive dependence of economic development on fossil fuels through technological innovation, new energy development, and the exploration of alternative energy sources. As a product of technological innovation and market demand driven by both directions, new energy vehicles to promote and apply clean energy will play an essential role in responding to energy crises and alleviating environmental pollution. Driven by dual carbon goals, developing new energy vehicles has gradually become a primary strategic choice for developing China’s automotive industry [26]. Therefore, it is necessary to conduct in-depth research on relevant issues related to the further development of the new energy vehicle industry.

In the existing literature, there is plenty of application of evolutionary game models to the research field of new energy vehicles. Among them, it is necessary to build an evolutionary game model between the government and automobile enterprises [27] and between automobile enterprises and consumers to analyze how to promote the sound diffusion of new energy vehicles (NEVs) [28]. Three parties combine the above three parties to build a three-party evolutionary game model of the government, automobile enterprises, and consumers to analyze the relevant issues of developing the new energy automobile industry [29]. In addition, some scholars have also applied the evolutionary game model to green technology innovation and green transformation of enterprises. Among them, the research on green technology innovation and green transformation of enterprises includes both the government and enterprises subjects [30], as well as the consideration of banks [31, 32] or the public [33, 34] as the third party subjects or the establishment of game models by bringing the above four parties into the same framework [35]. Among them, more and more scholars have introduced the four-party game model into the study of complex interaction mechanisms between different agents, including the study on the sustainability of green finance [36], the survey of policy-based financing to support green technology innovation of SMEs [37], and the study on the participation of asset management companies in corporate debt restructuring [38]. It can be seen that the four-party game model has particular applicability for solving complex problems between different stakeholders, and it also provides a specific reference for this article to introduce the four-party game model into the research on the strategy selection of different stakeholders in the development process of the new energy vehicle industry.

As an essential part of green finance, green credit is significant for promoting the real economy’s green and high-quality development [39]. The existing literature on green credit includes promoting green technological innovation of enterprises [40, 41], green total factor productivity [42–44], enterprise value [45], and its impact on the sustainable development of banks [46], etc. In addition, the research on green credit includes the control of the risk of green credit itself [47, 48] and evaluating the green credit efficiency of commercial banks based on the undesirable-SBM-DEA model [49]. Given the critical role of green credit in the development of the green economy, and in the context of the gradual decline of government subsidies, it is of great significance to introduce green credit to address the financial constraints faced by the development of the new energy vehicle industry and promote the healthy development of the new energy vehicle industry.

In summary, the existing literature on developing the new energy vehicle industry focuses on government subsidies and other perspectives, with less attention paid to the complementary role of green credit in government subsidies. Especially in the context of the gradual decline of government subsidies, the financial constraints faced by automobile manufacturers are becoming increasingly severe, and green credit will play a more significant role in developing the new energy vehicle industry. Secondly, the research on the development strategy selection of the new energy vehicle industry is mainly based on the interaction mechanism between the government and enterprises or between the government, enterprises, and consumers. Less attention is paid to the relationship between the game’s four players and less to the cost and benefit differences caused by asymmetric information between banks and enterprises in the model construction. In view of this, the marginal contribution of this study may lie in the following aspects: (1) Based on the issue that government subsidy adjustments have made automobile companies face increasingly severe financial constraints, this paper fully considers the complementary role of green credit in the decline of government subsidies, and analyzes its impact on the development of the new energy vehicle industry; (2) Different from previous bilateral and tripartite game analysis, this article, based on previous research, incorporates the government, automobile companies, banks, and consumers into the same game model framework, exploring the decision-making logic and equilibrium conditions of the four parties; (3) When establishing the game payment matrix of automobile enterprises and banking institutions, the signal transmission theory is creatively introduced, and the benefits and cost savings that can be brought to both parties by solving the information asymmetry problem between banks and enterprises are fully considered, so as to make the payment benefits of both parties’ entities more reasonable and more conducive to their optimal strategy selection.

## 2. Problem description and model construction

### 2.1 Problem description

The new energy vehicle industry has developed rapidly in recent years, driven by the national subsidy policy. However, with the adjustment of the national subsidy policy, the subsidy intensity for new energy vehicles is declining yearly, which may lead to an increase in the production costs of automobile enterprises. The financial constraints enterprises face will become increasingly severe, significantly impacting the development of the new energy vehicle industry. In response to the above issues, this article intends to adopt an evolutionary game approach, placing the government, automotive companies, banks, and consumers under the same analytical framework and studying the behavioral strategy selection of the four parties from the perspective of green credit and government subsidy adjustment. This study mainly discusses the following issues: ① Against the background of the rapid development of the new energy vehicle industry, how do changes in government subsidy strategies and subsidies affect the production of new energy vehicle enterprises? ② Considering the complementary role of green credit development in adjusting government subsidy policies, how does it affect the production strategy selection of new energy vehicle enterprises? How to guide consumers’ green consumption and its impact on developing the new energy vehicle industry?

**Fig 1:**
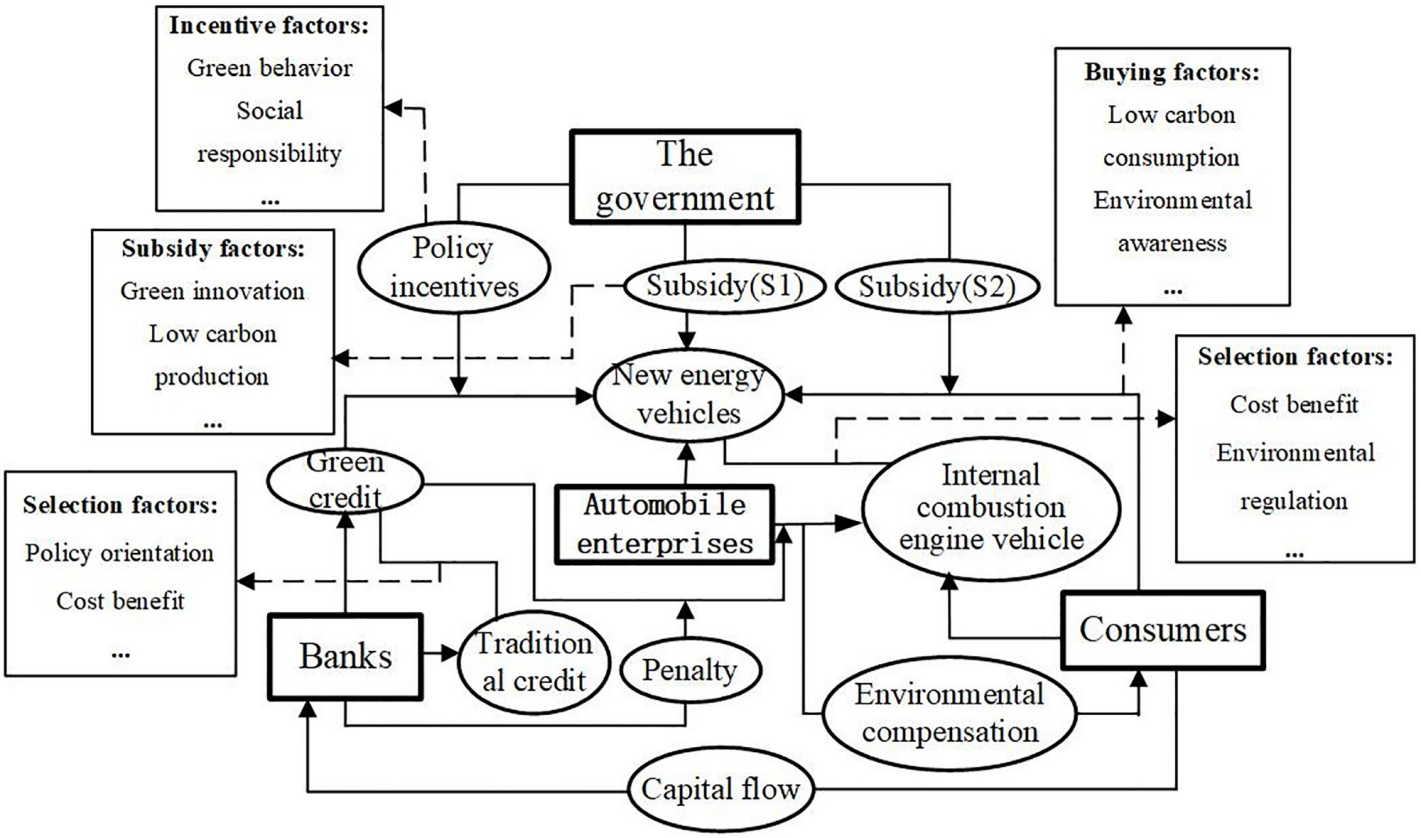
Logic relationship diagram of quadripartite subject decision.

This study constructs the decision-making logic relationship among the government, automobile companies, banks, and consumers, as shown in Fig 1.

From Fig 1, it can be seen that the government adopted a subsidy strategy to support the development of the new energy vehicle market. The subsidy includes purchase subsidy (*S*_1_) and purchase tax reduction (*S*_2_). Among them, *S*_1_ mainly acts on automobile enterprises and primarily works on low-carbon and *S*_2_ environmentally conscious consumers. Automobile companies mainly consider environmental regulation policies and cost-benefit factors when producing traditional internal combustion engines or new energy vehicles. Choosing to make new energy vehicles receives government subsidies and green credit support from banks.

Similarly, when deciding to make traditional internal combustion engine vehicles, it is not only impossible to obtain corresponding subsidies and green credit support but also necessary to pay additional environmental compensation. Banks will also punish automobile companies when there is "loan fraud" behavior. Banks choose traditional or green credit strategies based on policy orientation and cost-benefit factors. When banks choose green credit strategies, they are limited by the characteristics of long-term, high-risk, and relatively low-interest rates of green credit policies, resulting in low enthusiasm for implementing green credit. Therefore, government policy incentives are needed. For consumers, differences in environmental awareness and consumption concepts can lead to different consumption behaviors, and they may choose new energy vehicles or traditional internal combustion engine vehicles. The logical relationship between consumers and banks can be understood as consumers serving as essential providers of bank financial flows. With the increasing awareness of environmental protection among consumers, whether banks implement green credit strategies for consumers to evaluate the bank’s social responsibility will affect the provision of consumer financial flows [50].

### 2.2 Model building

To further study the strategic choices among various stakeholders in the development of the new energy vehicle industry, the following assumptions were made in the process of constructing a four-party stakeholder game model:

**Assumption 1**: This study chooses the government, automobile enterprises, banks, and consumers as the subjects of the game, and the four parties are all limited rational, which is more in line with the actual situation than the complete rationality of the traditional game. Each subject takes the maximization of interests as the goal of the game. As the leader in promoting high-quality economic development and protecting the environment, the government aims to maximize social benefits; auto enterprises and banks strive to maximize profits; as the main body of the consumer market, consumers seek to maximize their utility.
**Assumption 2**: ① The policy choice of the government is set as (subsidy or non-subsidy). The national subsidy for new energy vehicles mainly includes "automobile subsidy" and "automobile exemption from purchase tax". The primary purpose of subsidy strategies is to stimulate the implementation of phased policies for enterprise innovation, product promotion, and high-quality development. When the goals are achieved, they will gradually be eliminated. The probability of the government choosing subsidy strategies is *x*, and the probability of choosing de subsidy strategies is 1 − *x*. ② The strategic selection of automobile enterprises is set as (production, no production). The production strategy refers to the technology research and development of automobile enterprises or the introduction of advanced technology to produce new energy vehicles. The non-production strategy refers to producing internal combustion engine vehicles with traditional technology instead of new energy vehicles. Suppose the automobile enterprise’s probability of choosing the production strategy is *y*. In that case, the probability of choosing the non-production strategy is 1 − *y*.③ As the leading implementer of green credit, banking institutions’ strategic choices are (green credit and traditional credit). Banking institutions choose a green credit strategy with a *z* probability. Then the probability of traditional credit is 1 − *z*.④ As the main body of the consumer market, the consumer’s policy choice is set as (purchase, not purchase), where the consumer chooses the "purchase" strategy with a *w* probability. Then the probability of choosing the "no purchase" strategy is 1 − *w*.
**Assumption 3**: When the government chooses the "subsidy" strategy, it considers the production subsidy and carbon tax policy. The amount of government subsidies for the production of enterprises producing new energy vehicles is *S*_1_, and the vehicle purchase tax exempted for the purchase of new energy vehicles is *S*_2_; When the government grants subsidies to automobile enterprises, but the automobile enterprises choose to produce traditional internal combustion engine vehicles ("fraudulent compensation"), the government will recover the subsidies and punish the enterprises with a penalty coefficient of *θ*, then the penalty amount is *P*_*A*_ = *θS*_1_, and the automobile enterprises need to pay a carbon tax to the government *F*; When the banking institutions choose to implement the "green credit" policy, the government will give specific incentives, and the incentive coefficient is recorded as *λ*_1_ (*λ*_1_ ∈ [0,1]). When the "traditional credit" strategy is selected, the taxes to be paid are recorded as *T*_*B*_ and will be punished by the government. The penalty coefficient is recorded as *λ*_2_ (*λ*_2_ ∈ [0,1]).
**Assumption 4**: Technology level, government policies, consumer demand, and financial constraints affect an automobile enterprise’s strategic choice. Assuming that the net income of the enterprise from producing new energy vehicles is *R*_*NEV*_, the cost of technology research and development and production costs of the enterprise are recorded as *C*_*NEV*_, and the income from new energy vehicles is recorded as *r*_*NEV*_, then *R*_*NEV*_ = *r*_*NEV*_ − *C*_*NEV*_; When the net income of an automobile enterprise in traditional production is *R*_*ICEV*_, the production cost is *C*_*ICEV*_, and the income generated is recorded as *r*_*ICEV*_, then *R*_*ICEV*_ = *r*_*ICEV*_ − *C*_*ICEV*_ from the perspective of signal transmission (the core logic of signal transmission theory is that under the premise of information asymmetry, one party has no way to know the real value of the other party, so the party with information advantages can tell the party with information disadvantages its real value by sending a signal). When automobile enterprises produce new energy vehicles, they will receive government policy support, which to a certain extent, represents the government’s recognition, releases positive signals, and alleviates the problem of information asymmetry between enterprises and banks to a certain extent. At this time, enterprises are more likely to obtain the support of bank green credit, and the cost saving for enterprises (including time cost, opportunity cost, etc.) is recorded as *C*_*S*_. In addition, if the bank provides green credit to the enterprise but does not produce new energy vehicles ("fraudulent loan"), the bank will impose a fine on the enterprise and record it as *P*_*B*_.
**Assumption 5**: When a banking institution chooses to provide a "traditional credit" strategy for enterprises, the interest rate is recorded as *I*_*T*_, and the interest rate of the "green credit" strategy is recorded as *I*_*G*_. Assuming that the total credit amount of the banking institution is recorded as *CL*, where the green credit amount is *CL*_*G*_, and the traditional credit amount is *CL*_*T*_, the current green credit income is *R*_*G*_ = *CL*_*G*_ × *I*_*G*_ − *C*_*G*_, and the traditional credit income is R_*T*_ = *CL*_*T*_ × *I*_*T*_−*C*_*T*_. The bank’s cost of providing "green credit" is recorded as *C*_*G*_, including product research and development, employee training, product promotion, etc. The cost of "traditional credit" is recorded as *C*_*T*_. In addition, consumers with solid environmental awareness will pay more attention to the bank’s credit strategy choice. When the bank provides green credit, it will improve its reputation value and bring more customers to the bank to increase income. Currently, the additional income the bank obtains is recorded as Δ_*BB*_. However, when banks choose traditional credit rather than green credit business, once consumers with solid environmental awareness pay attention, they may lose this part of their customers. The additional losses incurred at this time are recorded as Δ_*SL*_. Due to the significant risk uncertainty of green credit investment, banks’ risk of green credit investment can be greatly reduced based on solving the information asymmetry between enterprises and banks through signal transmission theory. The additional income for banks is Δ_*E*_.
**Assumption 6**: When consumers choose the "purchase" strategy, the benefits are *R*_*C*1_; When consumers choose the "no purchase" strategy, the revenue from purchasing traditional internal combustion engine vehicles is *R*_*C*2_. However, implementing some restrictive measures has increased the use cost (record as *C*_*P*_) of traditional fuel vehicles (such as fuel tax, purchase tax, car lottery, etc.). Due to the inadequate supporting infrastructure, safety, and durability of new energy vehicles, the income from purchasing new energy vehicles is lower than that of traditional fuel vehicles, that is, *R*_*C*1_ < *R*_*C*2_. When an automobile enterprise obtains green credit and does not produce new energy vehicles, it must pay environmental compensation to the public, which is recorded as Δ_*EC*_.

Based on this, the payment matrix of government, automobile enterprises, banks, and consumers is shown in Table 1:

**Table 1:** Payment matrix of quartet players.

		Government subsidies(*x*)		Non-subsidy(1-*x*)	
Enterprise	Bank	Consumer purchase(*w*)	Non-purchase(1-*w*)	Purchase(*w*)	Non-purchase(1-*w*)
Production (*y*)	Green credit (*z*)	*a*_1_ = *λ*_1_*CL*_*G*_ − (*S*_1_ + *S*_2_)	*a*_2_ = *λ*_1_*CL*_*G*_ − *S*_1_	*a*_3_ = 0	*a*_4_ = 0
		*b*_1_ = *R*_*NEV*_ + *S*_1_ + *C*_*s*_	*b*_2_ = *S*_1_ + *C*_*S*_	*b*_3_ = *R*_*NEV*_ + *C*_*S*_	*b*_4_ = *C*_*S*_
		*c*_1_ = *R*_*G*_ + *λ*_1_*CL*_*G*_ + Δ_*E*_ + Δ_*BB*_	*c*_2_ = *R*_*G*_ + *λ*_1_*CL*_*G*_ + Δ_*E*_	*c*_3_ = *R*_*G*_ + *λ*_1_*CL*_*G*_ + Δ_*E*_ + Δ_*BB*_	*c*_4_ = *R*_*G*_ + *λ*_1_*CL*_*G*_
		*d*_1_ = *R*_*C*1_ + *S*_2_	*d*_2_ = *R*_*C*2_ − *C*_*P*_	*d*_3_ = *R*_*C*1_	*d*_4_ = *R*_*C*2_ − *C*_*P*_
	Traditional credit (1-*z*)	*a*_5_ = Δ_*EB*_ + λ_2_*CL*_*T*_ −(*S*_1_ + *S*_2_) + *T*_*B*_	*a*_6_ = Δ_*EB*_ + *λ*_2_*CL*_*T*_ − *S*_1_ + *T*_*B*_	*a*_7_ = Δ_*EB*_ + *λ*_2_*CL*_*T*_ + *T*_*B*_	*a*_8_ = Δ_*EB*_ + *λ*_2_*CL*_*T*_ + *T*_*B*_
		*b*_5_ = *R*_*NEV*_ + *S*_1_	*b*_6_ = *S*_1_	*b*_7_ = *R*_*NEV*_	*b*_8_ = 0
		*c*_5_ = *R*_*T*_ − *λ*_2_*CL*_*T*_ − *T*_*B*_ − Δ_*SL*_	*c*_6_ = *R*_*T*_ ‒ *λ*_2_*CL*_*T*_ − *T*_*B*_	*c*_7_ = *R*_*T*_ − *λ*2*CL*_*T*_ −*T*_*B*_ − Δ_*SL*_	*c*_8_ = *R*_*T*_ − *λ*_2_*CL*_*T*_ − *T*_*B*_
		*d*_5_ = *R*_*C*1_ + *S*_2_	*d*_6_ = *R*_*C*2_ − *C*_*P*_	*d*_7_ = *R*_*C*1_	*d*_8_ = *R*_*C*2_ − *C*_*P*_
Non- production (1-*y*)	Green credit (*z*)	*a*_9_ = *P*_*A*_ + *F* − *λ*_1_*CL*_*G*_	*a*_10_ = *P*_*A*_ + *F* − *λ*_1_*CL*_*G*_	*a*_11_ = *F* − *λ*_1_*CL*_*G*_	*a*_12_ = *F* − *λ*_1_*CL*_*G*_
		*b*_9_ = −*F* − *P*_*A*_ − *P*_*B*_ − Δ_*EC*_	*b*_10_ = *R*_*ICEV*_ − *F* − *P*_*A*_ − *P*_*B*_	*b*_11_ = −*F* − *P*_*B*_ − Δ_*EC*_	*b*_12_ = *R*_*ICEV*_ − *F* − *P*_*B*_
		*c*_9_ = *R*_*G*_ + *λ*_1_*CL*_*G*_ + Δ_*E*_ + Δ_*BB*_ + *P*_B_	*c*_10_ = *R*_*G*_ + *λ*_1_*CL*_*G*_ + Δ_*E*_ + *P*_*B*_	*c*_11_ = *R*_*G*_ + *λ*_1_*CL*_*G*_ + Δ_*BB*_ + *P*_B_	*c*_12_ = *R*_*G*_ + *λ*_1_*CL*_*G*_ + *P*_B_
		*d*_9_ = Δ_*EC*_	*d*_10_ = *R*_*C*2_ − *C*_*P*_	*d*_11_ = Δ_*EC*_	*d*_12_ = *R*_*C*2_ − *C*_*P*_
	Traditional credit (1-*z*)	*c*_13_ = *P*_*A*_ + *λ*_2_*CL*_*T*_ + *F*	*a*_14_ = *P*_*A*_ + *λ*_2_*CL*_*T*_ + *F*	*a*_15_ = *F* + *λ*_2_*CL*_*T*_	*a*_16_ = *F* + *λ*_2_*CL*_*T*_
		*b*_13_ = −*F* − *P*_*A*_	*b*_14_ = *R*_*ICEV*_ − *F* − *P*_*A*_	*b*_15_ = −*F*	*b*_16_ = *R*_*ICEV*_ − *F*
		*c*_13_ = *R*_*T*_ − *λ*_2_*CL*_*T*_ − *T*_*B*_ − Δ_*SL*_	*c*_14_ = *R*_*T*_ − *λ*_2_*CL*_*T*_ − *T*_*B*_	*c*_15_ = *R*_*T*_ − *λ*_2_*CL*_*T*_ − *T*_*B*_ − Δ_*SL*_	*c*_16_ = *R*_*T*_ − *λ*_2_*CL*_*T*_ − *T*_*B*_
		*d*_13_ = 0	*d*_14_ = *R*_*C*2_ − *C*_*P*_	*d*_15_ = 0	*d*_16_ = *R*_*C*2_ − *C*_*P*_

## 3. Analysis on the stability of the strategies of the game player

### 3.1 Stability analysis of government subsidy strategy

The expected return on the government’s choice of subsidy and non-subsidy strategies are *U*_*A*1_ and *U*_*A*2_, respectively, and the average expected return is *U*_*A*_. The dynamic replication equation and the first derivative of its behavior strategy are shown in Eqs (4) and (6).


$$\begin{array}{l} U_{A1}=yzwa_{1}+yz(1-w)a_{2}+y(1-z)wa_{5}+y(1-z)(1-w)a_{6}+(1-y)zwa_{9}\\ +(1-y)z(1-w)a_{10}+(1-y)(1-z)wa_{13}+(1-y)(1-z)(1-w)a_{14} \end{array}$$ (1)

UA2=yzwa3+yz(1−w)a4+y(1−z)wa7+y(1−z)(1−w)a8+(1−y)zwa11+(1−y)z(1−w)a12+(1−y)(1−z)wa15+(1−y)(1−z)(1−w)a16 (2)

$$U_{A}=xU_{A1}+(1-x)U_{A2}$$ (3)

$$F(x)=x(U_{A1}-U_{U_{A}})=x(1-x)(U_{A1}-U_{A2})=x(1-x)f(y,z,w)$$ (4)

$$f(y,z,w)=P_{A}-y(P_{A}+S_{1}+z\lambda_{1}CL_{G}+wS_{2})$$ (5)

$$F^{\prime }(x)=(1-2x)f(y,z,w)$$ (6)

According to the stability theorem of a differential equation, the policy choice of the government in a stable state should meet the following requirements: *F*(*x*) = 0 and *F*′(*x*) = 0.

**Proposition 1**:

- When *y* < *y*_0_, the government’s stability strategy is "subsidy".
- When *y* > *y*_0_, the stable strategy is " non-subsidy ".
- When *y* = *y*_0_, the government’s stabilization strategy cannot be determined. The threshold value is *y*_0_ = *P*_*A*_/(*P*_*A*_ + *S*_1_ + *zλ*_1_*CL*_*G*_ + *wS*_2_).

**Proof**: Since *∂f*(*y*,*z*,*w*)/*∂y* = −(*P*_*A*_ + *S*_1_ + *zλ*_1_*CL*_*G*_ + *wS*_2_) < 0, *f*(*y*,*z*,*w*) is a decreasing function for *y*. When *y* < *y*_0_, there are *f*(*y*,*z*,*w*) > 0, *F*(*x*)|_*x* = 1_ = 0, and *F*′(*x*)|_*x* = 1_ = 0, where *x* = 1 has stability; When *y* > *y*_0_, there are *f*(*y*,*z*,*w*) < 0, *F*(*x*)|_*x* = 0_ = 0, and *F*′(*x*)|_*x* = 0_ < 0, where *x* = 0 has stability; When *y* = *y*_0_, there are *F*(*x*) = 0 and *F*′(*x*) = 0, where the stability strategy cannot be determined, and it is proved.

Proposition 1 indicates that in the government’s strategic choice, if the enterprise’s willingness to produce new energy vehicles is low, the government’s strategic choice will be a "subsidy" strategy. In the context of the in-depth implementation of the basic national environmental protection policy, the state has mobilized the enthusiasm of automobile enterprises to produce new energy vehicles through policy regulation to achieve sustainable development. As the willingness of automobile enterprises to make new energy vehicles gradually increases and exceeds the threshold, the willingness of automobile enterprises to produce new energy vehicles is relatively strong, and the government’s subsidy intensity will decline year by year. It will be canceled when the mission is ultimately completed. This is also in line with China’s subsidy policy change trend. Therefore, as the willingness of automobile manufacturers to produce new energy vehicles gradually increases, the government’s subsidies will gradually decrease and eventually be eliminated.

**Fig 2:**
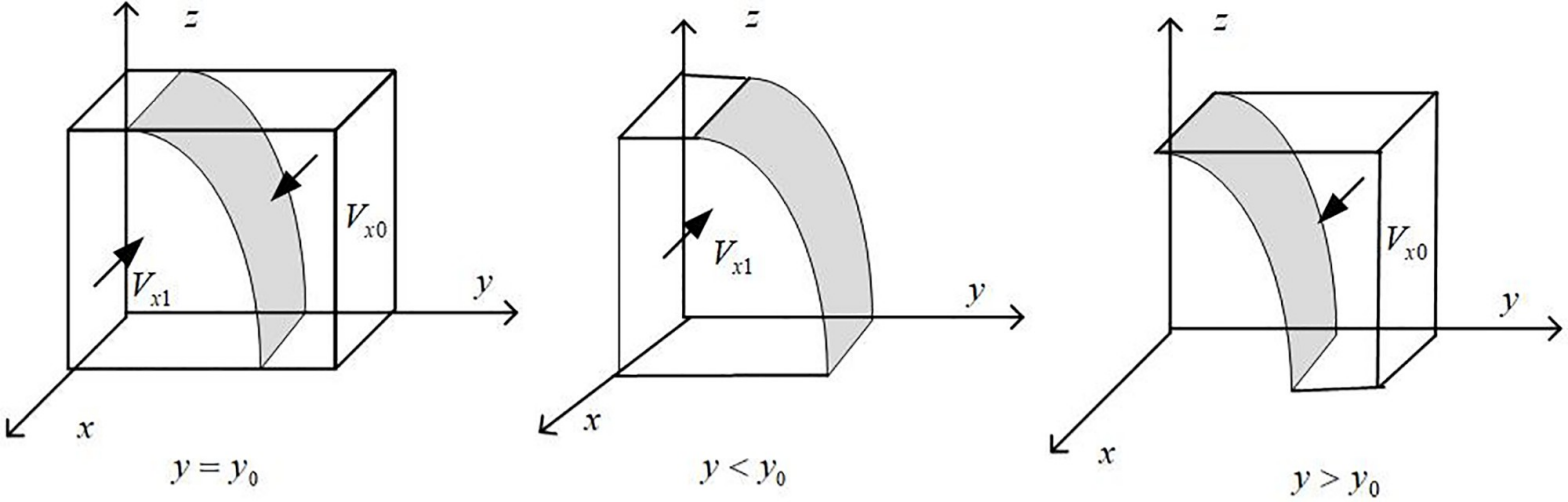
Phase diagram of government strategy selection.

The phase diagram of government strategy selection drawn according to Proposition 1 is shown in Fig 2:

It can be seen from Fig 2 that the volume of part *V*_*x*1_ is the probability of the government choosing "subsidy", and the volume of part *V*_*x*0_ is the probability of the government choosing " non-subsidy ". It is calculated that:

$$V_{x1}=\int \begin{array}{l} 1\\ 0 \end{array}\int \begin{array}{l} 1\\ 0 \end{array}[P_{A}/(P_{A}+S_{1}+z\lambda_{1}CL_{G}+wS_{2})]dzdx=P_{A}\;\text{ln}[1+\lambda_{1}CL_{G}/(P_{A}+S_{1}+wS_{2})]/\lambda_{1}CL_{G}$$ (7)

Then there are:

$$V_{x0}=1-V_{x1}=1-P_{A}\;\text{ln}[1+\lambda_{1}CL_{G}/(P_{A}+S_{1}+wS_{2})]/\lambda_{1}CL_{G}$$ (8)

**Inference 1.1**: As the probability of consumers purchasing new energy vehicles and the punishment for "fraudulent subsidy" behavior of automobile companies gradually increases, that is, the probability of "fraudulent subsidy" behavior in the new energy vehicle market with good sales prospects and leading to market chaos will gradually decrease, and the government’s strategic choice will shift progressively from "subsidies" to "de subsidies". There is a threshold value for the government’s subsidy strategy. Before reaching the threshold value, the subsidy intensity will gradually increase. Still, when exceeding a specific threshold value, the effectiveness of government subsidies will gradually decrease, so the government will gradually reduce and eventually cancel subsidies. The government will shift from a "subsidy" strategy to a "de-subsidy" strategy.

**Proof**: Calculate the first derivative of *V*_*x*1_ for *w* and *P*_*A*_ to obtain: *∂V*_*x*1_/*∂*_*w*_ < 0, *∂V*_*x*1_/*∂P*_*A*_ < 0. It can be seen that *V*_*x*1_ is negatively correlated with *w* and *P*_*A*_. According to the preceding, let *f*(*S*_1_) = *P*_*A*_−*y*(*P*_*A*_ + *S*_1_ + *zλ*_1_*CL*_*G*_ + *wS*_2_), we can see *∂f*/*∂S*_1_ < 0, and we can see that *f* is the decreasing function of *S*_1_, where *S*_0_ = [*P*_*A*_−*y*(*P*_*A*_ + *zλ*_1_*CL*_*G*_ + *wS*_2_)]/*y*. When *S*_1_ > *S*_0_, there are *F*(*x*)|_*x* = 0_ = 0 and *F*′(*x*)|_*x* = 0_ < 0. Currently, the government’s strategic choice tends to be a "non-subsidy". When *S*_1_ < *S*_0_, there are *F*(*x*)|_*x* = 1_ = 0 and *F*′(*x*)|_*x* = 1_ < 0. Currently, the government’s strategic choice tends to be a "subsidy" strategy. Therefore, there is a threshold for government subsidies for new energy vehicles. In the early stage, the government’s willingness to subsidize will increase as the subsidy intensity increases. However, after reaching the threshold, the government subsidies will gradually decrease and eventually change from "subsidies" to a "de subsidies" strategy.

### 3.2 Analysis of strategic stability of automobile enterprises

The expectation of the automobile enterprise to choose the "production" and "non-production" strategies is *U*_*B*1_ and *U*_*B*2_, respectively, and the average income is *U*_*B*_. The dynamic replication equation and the first derivative of its behavior strategy are shown in Eqs (12) and (14).


U=B1xzwb1+xz(1−w)b2+x(1−z)wb5+x(1−z)(1−w)b6+(1−x)zwb3+(1−x)z(1−w)b4+(1−x)(1−z)wb+7(1−x)(1−z)(1−w)b8 (9)

$$\begin{array}{l} U_{B2}=xzwb_{9}+xz(1-w)b_{10}+x(1-z)wb_{13}+x(1-z)(1-w)b_{14}+(1-x)zwb_{11}\\ +(1-x)z(1-w)b_{12}+(1-x)(1-z)wb_{15}+(1-x)(1-z)(1-w)b_{16} \end{array}$$ (10)

$$U_{B}=yU_{B1}+(1-y)U_{B2}$$ (11)

$$F(y)=y(U_{B1}-U_{B})=y(1-y)(U_{B1}-U_{B2})=y(1-y)f_{1}(x,z,w)$$ (12)

$$f_{1}(x,z,w)=F-R_{ICEV}+z(C_{S}+P_{B})+x(P_{A}+S_{1})+w(R_{ICEV}+R_{NEV}+z\Delta_{EC})$$ (13)

$$F^{\prime }(y)=(1-2y)f_{1}(x,z,w)$$ (14)

According to the stability theorem of the differential equation, the strategy of automobile enterprises in a stable state should meet the following requirements: *F*(*y*) = 0 and *F*′(*y*) < 0.

**Proposition 2**:

- When *w* < *w*_0_, automobile enterprises tend to produce new energy vehicles.
- When *w* > *w*_0_, the strategic choice of automobile enterprises tends to be a "non-production" strategy.
- When *w* = *w*_0_, the stable strategy of the automobile enterprise cannot be determined. The threshold is *w*_0_ = [*R*_*ICEV*_−*F*–*z*(*C*_*S*_ + *P*_*B*_)–*x*(*P*_*A*_ + *S*_1_)]/(*R*_*ICEV*_ + *R*_*NEV*_ + *z*Δ_*EC*_).

**Proof**: It can be seen from *∂f*_1_(*x*,*z*,*w*)/*∂*_*w*_ > 0 that *f*_1_(*x*,*y*,*w*) is an increasing function of *w*. When *w* < *w*_0_, there are *f*_1_(*x*,*z*,*w*) < 0, *F*(*y*)|_*y* = 0_ = 0, and *F*′(*y*)|_*y* = 0_ < 0, where *y* = 0 is stable; When *w* > *w*_0_, there are *f*_1_(*x*,*z*,*w*) > 0, *F*(*y*)|_*y* = 1_ = 0, and *F*′(*y*)|_*y* = 1_ < 0, where *y* = 1 is stable; When *w* = *w*_0_, *F*(*y*) = 0, and *F*(*y*′) = 0, and the stability cannot be determined at this time, which is verified.

Proposition 2 indicates that the choice of whether to produce new energy vehicles by automotive companies is closely related to consumers’ consumption beliefs. When consumers have a relatively low willingness to purchase new energy vehicles, the strategic choice of automotive companies is to "not produce". However, as consumers’ consumption concepts change and their awareness of environmental protection increases, they are more inclined to purchase new energy vehicles, and the strategic choice of automotive companies will shift from "not producing" to "producing", which is also consistent with the actual situation of the new energy vehicle market.

**Fig 3:**
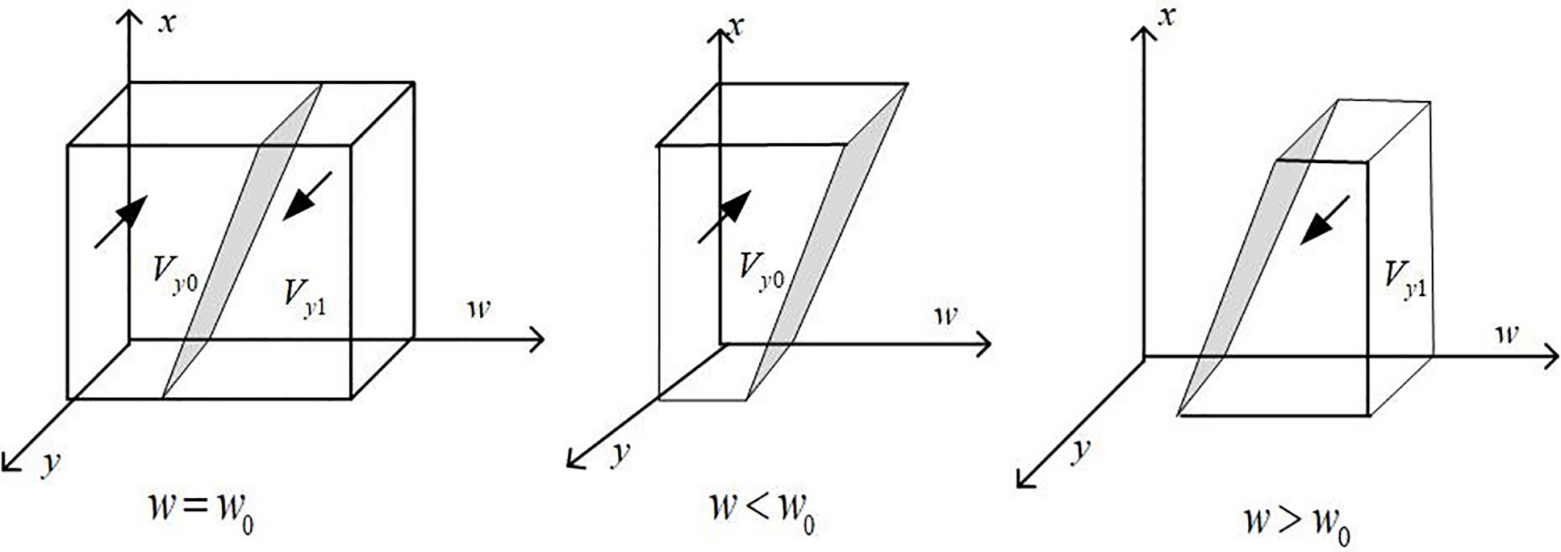
Phase diagram of strategy selection of automobile enterprises.

Draw the phase diagram of automobile enterprise strategy selection according to Proposition 2, as shown in Fig 3:

It can be seen from Fig 3 that the volumes of the *V*_*y*0_ and *V*_*y*1_ parts are the probabilities of the automobile enterprises to choose the production and non-production strategies, respectively, and it is calculated that:

$$\begin{array}{ll} V_{y0} & =\int \begin{array}{l} 1\\ 0 \end{array}\int \begin{array}{l} 1\\ 0 \end{array}[R_{ICEV}-F-z(C_{S}+P_{B})-x(P_{A}+S_{1})]/(R_{ICEV}+R_{NEV}+z\Delta_{EC})dxdy\\ & =-(2F+P_{A}-2R_{ICEV}+S_{1}+2zC_{S}+2zP_{B})/2(R_{ICEV}+R_{NEV}+z\Delta_{EC}) \end{array}$$ (15)

Then there are:

$$V_{y1}=1+(2F+P_{A}-2R_{ICEV}+S_{1}+2zC_{S}+2zP_{B})/2(R_{ICEV}+R_{NEV}+z\Delta_{EC})$$ (16)

**Inference 2.1**: When automotive companies receive government subsidies and green credits from banking institutions without producing new energy vehicles, the penalties they face, the carbon tax they need to pay to produce traditional internal combustion engine vehicles, the subsidies provided by the government, and the resulting signal effect increase the cost savings for the company, automotive companies will be more inclined to choose to produce new energy vehicles; When the net income from producing traditional internal combustion engine vehicles increases, automotive companies are more inclined to choose to produce internal combustion engine vehicles rather than new energy vehicles.

**Proof**: Calculate the first derivative of the probability *V*_*y*1_ of an automobile enterprise choosing to produce new energy vehicles for *P*_*A*_, *S*_1_, *C*_*S*_, *P*_*B*_ and *F* to obtain: *∂V*_*y*1_/*∂P*_*A*_>0, *∂V*_*y*1_/*∂S*_1_ > 0, *∂V*_*y*1_/*∂C*_*S*_ > 0, *∂V*_*y*1_/*∂P*_*B*_ > 0, *∂V*_*y*1_/*∂F* < 0. From this, it can be seen that *V*_*y*1_ is positively correlated with *P*_*A*_, *S*_1_, *C*_*S*_, *P*_*B*_, and negatively correlated with *F*. The certificate is completed.

**Inference 2.2**: The government subsidizes automobile enterprises to produce new energy vehicles. Banking institutions provide green credit to enterprises to help them solve financial constraints in developing and producing new energy vehicles. When automobile enterprises receive subsidies and green capital support and do not produce new energy vehicles, it will harm the entire economy and society. To address the possible "fraudulent subsidy" and "loan fraud" behaviors of automobile enterprises, the required dual penalties of the government and banks must reach a certain threshold. Only when the amount of punishment comes to a certain threshold can it be possible to avoid the above behaviors of automobile enterprises?

**Proof**: Let *f*_1_(*P*_*A*_) = *F*–*R*_*ICEV*_ + *z*(*C*_*S*_ + *P*_*B*_) + *x*(*P*_*A*_ + *S*_1_) + *w*(*R*_*ICEV*_ + *R*_*NEV*_), then *∂f*_1_(*P*_*A*_)/*∂P*_*A*_>0, we can see that *f*_1_ is an increasing function of *P*_*A*_, where $P_{A}^{*}=[R_{ICEV}-F-xS_{1}-z(C_{S}+P_{B})-w(R_{ICEV}+R_{NEV})]/x$. When $P_{A}>P_{A}^{*}$, there are *F*(*y*)|_*y* = 1_ = 0 and *F*′(*y*)|_*y* = 1_ < 0, and the automobile enterprise’s strategic choice is to produce new energy vehicles. Similarly, when $P_{A}<P_{A}^{*}$, there are *F*(*y*)|_*y* = 0_ = 0 and *F*′(*y*)|_*y* = 0_ < 0, and the automobile enterprise’s strategic choice is not to produce new energy vehicles. *P*_*B*_ is provable by the same reasoning. It can be seen from this that before the government and the bank’s punishment reaches the threshold. Automobile enterprises have a fluke mentality and will not produce new energy vehicles. However, when the punishment value exceeds a certain threshold, automobile enterprises that receive subsidies and green funds will likely make new energy vehicles.

### 3.3 Stability analysis of bank strategy choice

The expected returns of banks implementing green and traditional credit strategies are *U*_*C*1_ and *U*_*C*2_, respectively, and the average expected return is *U*_*C*_. The dynamic replication equation and the first derivative of its behavior strategy are shown in Eqs (20) and (22).


$$\begin{array}{l} U_{C1}=xywc_{1}+xy(1-w)c_{2}+(1-x)ywc_{3}+(1-x)y(1-w)c_{4}+x(1-y)wc_{9}\\ +x(1-y)(1-w)c_{10}+(1-x)(1-y)wc_{11}+(1-x)(1-y)(1-w)c_{12} \end{array}$$ (17)

$$\begin{array}{l} U_{C2}=xywc_{5}+xy(1-w)c_{6}+(1-x)ywc_{7}+(1-x)y(1-w)c_{8}+x(1-y)wc_{13}\\ +x(1-y)(1-w)c_{14}+(1-x)(1-y)wc_{15}+(1-x)(1-y)(1-w)c_{16} \end{array}$$ (18)

$$U_{C}=zU_{C1}+(1-z)U_{C2}$$ (19)

$$F(z)=z(U_{C1}-U_{C})=z(1-z)(U_{C1}-U_{C2})=z(1-z)f_{2}(x,y,w)$$ (20)

$$f_{2}(x,y,w)=(1-y)P_{B}+w(\Delta_{SL}+\Delta_{BB})+x\Delta_{E}+\lambda_{1}CL_{G}+\lambda_{2}CL_{T}+R_{G}-R_{T}+T_{B}$$ (21)

$$F^{\prime }(z)=(1-2z)f_{2}(x,y,w)$$ (22)

According to the stability theorem of a differential equation, the strategy of a banking institution in a stable state shall meet the following requirements: *F*(*z*) = 0 and *F*′(*z*) < 0.

**Proposition 3**:

- When *x* < *x*_1_, *y* > *y*_1_, *w* < *w*_1_,the strategic choice of banking institutions is to implement traditional credit strategies.
- When *x* > *x*_1_, *y* < *y*_1_, *w* > *w*_1_, the banking institution’s strategy is to implement a green credit strategy.
- When *x* = *x*_1_, *y* = *y*_1_, *w* = *w*_1_, the stability strategy of the banking institution cannot be determined.

Among them:

$$x_{1}=[R_{T}-R_{G}-T_{B}-\lambda_{1}CL_{G}-\lambda_{2}CL_{T}-(1-y)P_{B}-w(\Delta_{SL}+\Delta_{BB})]/\Delta_{E}$$

$$y_{1}=[P_{B}-R_{T}+R_{G}+T_{B}+\lambda_{1}CL_{G}+\lambda_{2}CL_{T}+w(\Delta_{SL}+\Delta_{BB})+x\Delta_{E}]/P_{B}$$

$$w_{1}=[R_{T}-R_{G}-T_{B}-\lambda_{1}CL_{G}-\lambda_{2}CL_{T}-(1-y)P_{B}-x\Delta_{E}]/(\Delta_{SL}+\Delta_{BB})$$

**Proof**: Since *∂f*_2_(*x*, *y*, *w*)/*∂x* > 0, *f*_1_(*x*, *y*, *w*) is an increasing function of *x*. When *x* < *x*_1_, there are *f*_2_(*x*, *y*, *w*) < 0, *F*(*z*)|_*z* = 0_ = 0, and *F*′(*z*)|_*z* = 0_ < 0, and *z* = 0 is stable; When *x* > *x*_1_, there are *f*_2_(*x*, *y*, *w*) < 0, *F*(*z*)|_*z* = 1_ = 0, and *F*′(*z*)|_*z* = 1_ < 0, and *z* = 1 is stable; When *x* = *x*_1_, *F*(*z*) = 0 and *F*′(*z*) = 0, and stability strategy of the banking institution cannot be determined at this time. In addition, since *∂f*_2_(*x*, *y*, *w*)/*∂*_*y*_ < 0 and *∂f*_2_(*x*, *y*, *w*)/*∂*_*w*_ > 0, it can be seen that *f*_1_(*x*, *y*, *w*) is a decreasing function of *y* and the increasing function of *w*. The other proofs are the same as the above proofs of *x*, and the certificate is completed.

Proposition 3 indicates that when the probability of government subsidies and consumers’ purchase intentions are low, even automotive companies have a solid willingness to produce new energy vehicles. However, due to the signal effect, banks will choose traditional credit instead of taking risks to provide green credit while complying with government strategies and taking into account the consumer market prospects of new energy vehicles; As the government’s willingness to subsidize and consumers’ willingness to purchase new energy vehicles gradually increases, even though the production willingness of new energy vehicle companies is relatively low at this time, due to the guidance of national industrial policies and the optimistic market prospects of new energy vehicles, banks’ strategic choices will shift from traditional credit strategies to green credit strategies. With the development of the economy, Green credit will become the mainstream policy choice for most banking institutions.

**Fig 4:**
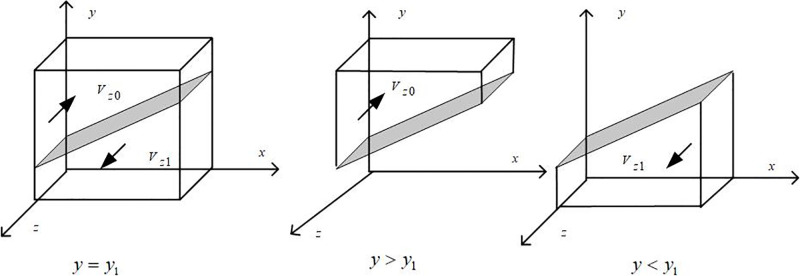
Phase diagram of strategy selection of banking institutions.

Draw the phase diagram of banking institution strategy selection according to Proposition 3, as shown in Fig 4.

It can be seen from Fig 4 that the volume of part *V*_*z*0_ and part *V*_*z*1_ is the probability of banking institutions choosing traditional and green credit, respectively, and it is calculated that:

$$\begin{array}{ll} V_{z1} & =\int \begin{array}{l} 1\\ 0 \end{array}\int \begin{array}{l} 1\\ 0 \end{array}\{[P_{B}-R_{T}+R_{G}+T_{B}+\lambda_{1}CL_{G}+\lambda_{2}CL_{T}+w(\Delta_{SL}+\Delta_{BB})+x\Delta_{E}]/P_{B}\}dxdz\\ & =[[\Delta_{E}/2+\lambda_{1}CL_{G}+\lambda_{2}CL_{T}+R_{G}-R_{T}+T_{B}+w(\Delta_{SL}+\Delta_{BB})]/P_{B}]+1 \end{array}$$ (23)

Then there are:

$$V_{z0}=1-V_{z1}=-[[\Delta_{E}/2+\lambda_{1}CL_{G}+\lambda_{2}CL_{T}+R_{G}-R_{T}+T_{B}+w(\Delta_{SL}+\Delta_{BB})]/P_{B}]$$ (24)

**Inference 3.1**: With the government’s incentives for banking institutions, penalties for traditional credit, taxes, and fees to be paid, green credit income, and the additional income it can bring, banks will be more inclined to implement green credit strategies; When the income brought by traditional credit and the amount of punishment that can be obtained by providing green credit to enterprises without producing new energy vehicles are reduced, banks tend to choose traditional credit policies.

**Proof**: The first partial derivative of the probability of the bank implementing the green credit policy *V*_*z*1_ versus Δ_*E*_, *λ*_1_*CL*_*G*_, *λ*_2_*CL*_*T*_, *R*_*G*_, *R*_*T*_, *T*_*B*_, Δ_*SL*_, Δ_*BB*_, and *P*_*B*_ is obtained: *∂V*_*z*1_/*∂*Δ_*E*_ > 0, ∂*V*_*z*1_/*∂R*_*G*_ > 0, *∂V*_*z*1_/*∂R*_*T*_ < 0, etc. The same reason as the above can prove the rest.

**Inference 3.2**: There are thresholds for the government’s incentives (*λ*_1_*CL*_*G*_) to implement green credit policies for banks and for the punishment (*λ*_2_*CL*_*T*_) to choose traditional credit strategies. When below the threshold, banks tend to prefer traditional credit policies; When the punishment and incentive intensity exceeds the threshold, the strategic choice of banks will gradually shift from traditional credit to green credit.

**Proof**: Assuming *m* = *λ*_1_*CL*_*G*_ and *n* = *λ*_2_*CL*_*T*_, we have *f*_2_(*m*,*n*) = (1 − *y*)*P*_*B*_ + *w*(Δ_*SL*_ + Δ_*BB*_) + *x*Δ_*E*_ + *m* +*n* + *R*_*G*_−*R*_*T*_ + *T*_*B*_. We can get *∂f*_2_(*m*,*n*)/*∂*_*m*_ > 0, *∂f*_2_(*m*,*n*)/*∂n* > 0, so we can know that *f*_2_(*m*,*n*) is an increasing function of *m* and *n*, where:

$$m_{1}=R_{T}-R_{G}-T_{B}-n-(1-y)P_{B}-w(\Delta_{SL}+\Delta_{BB})-x\Delta_{E}$$

$$n_{1}=R_{T}-R_{G}-T_{B}-m-(1-y)P_{B}-w(\Delta_{SL}+\Delta_{BB})-x\Delta_{E}$$

When *m* < *m*_1_, there are *F*(*z*)|_*z* = 0_ = 0 and *F*′(*z*)|_*z* = 0_ < 0, where *z* = 0 is stable; When *m* > *m*_1_, there are *F*(*z*)|_*z* = 1_ = 0 and *F*′(*z*)|_*z* = 1_ < 0, where *z* = 1 is stable. In the same way, it can be proved that *n* meets the above conditions.

### 3.4 Stability analysis of consumer strategy selection

The expected return of consumers’ choice to buy and not to purchase strategies is *U*_*D*1_ and *U*_*D*2_, respectively, and the average expected return is *U*_*D*_. The dynamic replication equation and the first derivative of their behavior strategies are shown in Eqs (28) and (30).


$$\begin{array}{l} U_{D1}=xyzd_{1}+xy(1-z)d_{5}+(1-x)yzd_{3}+(1-x)y(1-z)d_{7}+x(1-y)zd_{9}\\ +x(1-y)(1-z)d_{13}+(1-x)(1-y)zd_{11}+(1-x)(1-y)(1-z)d_{15} \end{array}$$ (25)

$$\begin{array}{l} U_{D2}=xyzd_{2}+xy(1-z)d_{6}+(1-x)yzd_{4}+(1-x)y(1-z)d_{8}+x(1-y)zd_{10}\\ +x(1-y)(1-z)d_{14}+(1-x)(1-y)zd_{12}+(1-x)(1-y)(1-z)d_{16} \end{array}$$ (26)

$$U_{D}=wU_{D1}+(1-w)U_{D2}$$ (27)

$$F(w)=w(U_{D1}-U_{D})=w(1-w)(U_{D1}-U_{D2})=f_{3}(x,y,z)$$ (28)

$$f_{3}(x,y,z)=C_{P}-R_{C2}+(1-y)z\Delta_{EC}+yR_{C1}+xyS_{2}$$ (29)

$$F^{\prime }(w)=(1-2w)f_{3}(x,y,z)$$ (30)

The main influencing factors of consumers’ strategic choice are the benefits of purchasing two types of cars and the government’s subsidy intensity *S*_2_. According to the stability theorem of the differential equation, the consumer’s strategy needs to meet the following conditions: *F*(*w*) = 0 and *F*′(*w*) < 0.

**Proposition 4**:

- When *x* > *x*_2_, *z* > *z*_2_, the stable strategy of consumers is to buy new energy vehicles.
- When *x* < *x*_2_, *z* < *z*_2_, the consumer’s stability strategy is not to buy new energy vehicles but to choose traditional internal combustion engines.
- When *x* = *x*_2_, *z* = *z*_2_, the consumer’s stability strategy cannot be determined where the threshold is:

$$x_{2}=[R_{C2}-C_{P}-(1-y)z\Delta_{EC}-yR_{C1}]/yS_{2}$$

$$z_{2}=[C_{P}-R_{C2}+yR_{C1}+xyS_{2}]/(y-1)\Delta_{EC}$$

**Proof**: Since *∂f*_3_(*x*, *y*, *z*)/∂_*x*_ > 0,then *f*_3_(*x*, *y*, *z*) is an increasing function of *x*. When *x* > *x*_2_, there are *f*_3_(*x*, *y*, *z*) > 0, *F*(*w*)|_*w* = 1_ = 0, and *F*′(*w*)|_*w* = 1_ < 0, then *w* = 1 is stable, and consumers tend to buy new energy vehicles; When *x* < *x*_2_, there are *f*_3_(*x*, *y*, *z*) < 0, *F*(*w*)|_*w* = 0_ = 0, and *F*′(*w*)|_*w* = 0_ < 0, then *w* = 0 is stable, and consumers tend to choose traditional internal combustion engines instead of new energy vehicles. It can be proved that the above conditions are also met *z*, and the certificate is completed.

Proposition 4 indicates that when government subsidies for new energy vehicles are relatively small, and banking institutions have no significant bias towards implementing green credit policies, consumers will have more substantial uncertainty about the new energy vehicle market. The possibility of purchasing new energy vehicles is also low. However, as the intensity of government subsidies gradually increases and the probability of banking institutions providing green credit increases, consumers will slowly change their attitudes towards the new energy vehicle market when considering the national industrial policy orientation and changes in the strategic behavior of banking institutions. At this time, consumers’ strategic choices tend to purchase new energy vehicles.

**Fig 5:**
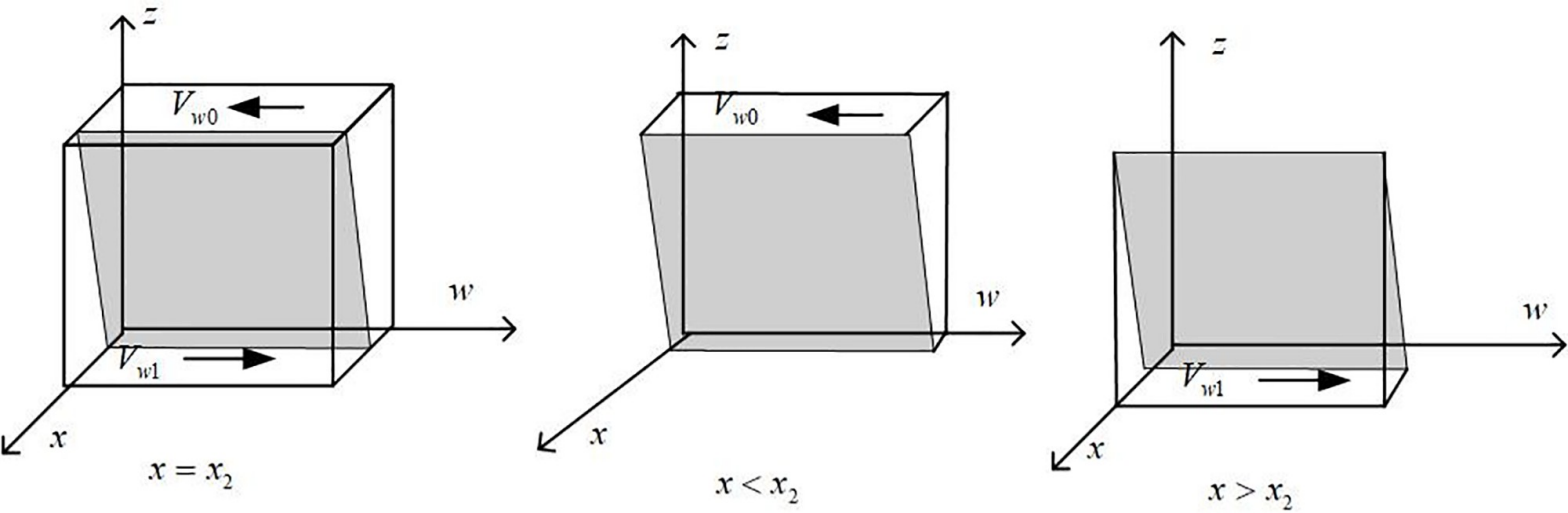
Phase diagram of consumer strategy selection.

Draw the phase diagram of consumer strategy selection according to the above proposition, as shown in Fig 5:

It can be seen from Fig 5 that the volume of part *V*_*w*0_ and part *V*_*w*1_, respectively, represents the probability that consumers will not buy new energy vehicles and purchase new energy vehicles, which is calculated as follows:

$$\begin{array}{ll} V_{w0} & =\int \begin{array}{l} 1\\ 0 \end{array}\int \begin{array}{l} 1\\ 0 \end{array}[[R_{C2}-C_{P}-(1-y)z\Delta_{EC}-yR_{C1}]/yS_{2}]dzdw\\ & =[y(\frac{1}{2}\Delta_{EC}-R_{C1})-C_{P}-\frac{1}{2}\Delta_{EC}+R_{C2}]/(yS_{2}) \end{array}$$ (31)

Then there are:

$$V_{w1}=1-V_{w0}=1-[y(\frac{1}{2}\Delta_{EC}-R_{C1})-C_{P}-\frac{1}{2}\Delta_{EC}+R_{C2}]/(yS_{2})$$ (32)

**Inference 4.1**: With the increase of the benefits of new energy vehicles, subsidies for the purchase of new energy vehicles, and the cost of traditional internal-combustion engine vehicles, and the decrease of the benefits of the purchase of traditional internal-combustion engine vehicles, the probability of consumers buying new energy vehicles will also increase, and vice versa.

**Proof**: It is obtained by calculating the first partial derivative of the probability *V*_*w*1_ of consumers purchasing new energy vehicles for *R*_*C*1_, *R*_*C*2_, *S*_2_, Δ_*EC*_, and *C*_*P*_. We can get ∂*V*_*w*1_/∂*R*_*c*1_ > 0, ∂*V*_*w*1_/∂*S*_2_ > 0 (the conditions are *R*_*C*2_ − *C*_*P*_ > *yR*_*C*1_ + 1/2(1 − *y*)Δ_*EC*_), ∂*V*_*w*1_/∂Δ_*EC*_ > 0, ∂*V*_*w*1_/∂*C*_*P*_ > 0, ∂*V*_*w*1_/∂*R*_*C*2_ < 0. It can be seen that the probability of consumers buying new energy vehicles is positively correlated with *R*_*C*1_, Δ_*EC*_, *S*_2_, *C*_*P*_, and negatively correlated with *R*_*C*2_.

**Inference 4.2**: If the conditions are met, the income from purchasing a traditional internal combustion engine vehicle is higher than that from new energy vehicles. The government needs to take measures, such as increasing the cost of traditional internal combustion engine vehicles and increasing subsidies for new energy vehicles. However, consumers have a subsidy threshold to purchase new energy vehicles. When *S*_2_ is greater than threshold *S*_2_*, consumers’ willingness to purchase new energy vehicles increases. Consumers tend only to buy the strategy when the subsidy *S*_2_ is at least the threshold *S*_2_*.

**Proof**: If *f*_3_ (*S*_2_) = *C*_*P*_ − *R*_*C*2_ + (1 − *y*)*z*Δ_*EC*_ + *yR*_*C*1_ + *xyS*_2_, we can calculate ∂*f*_3_ (*S*_2_)/*∂S*_2_ > 0 and know that *f*_3_ (*S*_2_) is an increasing function of *S*_2_. Therefore, when *S*_2_ < *S*_2_*, there are *F*(*w*)|_*w* = 0_ = 0 and *F*′(*w*)|_*w* = 0_ < 0, and *x* = 0 is stable. At this time, consumers tend to choose not to buy new energy vehicles; when *S*_2_ > *S*_2_*, there are *F*(*w*)|_*w* = 1_ = 0 and *F*′(*w*)|_*w* = 1_ < 0, and *x* = 1 is stable, the probability of consumers buying new energy vehicles increases. Where *S*_2_* = [*R*_*C*2_ − *C*_*P*_ − (1 − *y*)*z*Δ_*EC*_ − *yR*_*C*1_]/*xy*, the certificate is completed.

## 4. Stability analysis of strategy combination

### 4.1 An analysis of the equilibrium point of the four-way evolutionary game of government-auto enterprise-bank-consumer

Under the background of high-quality development, to explore the conditions and formation process of the strategy selection of different entities in the development of the new energy automobile industry, in the construction of a dynamic replication system of the four-party game of government, automobile enterprises, banks, and consumers, the stability of the strategy group of the four-party entities can be judged by Lyapunov’s first rule. Ritzberger [51] and Selten [52] pointed out that in the multi-group evolutionary game, the stable solution of the evolutionary game is the strict Nash equilibrium, and the strict Nash equilibrium is a pure strategy. Therefore, this paper only analyzes the stability of 16 groups of pure strategy equilibrium solutions and constructs the Jacobian matrix of the four-party agent replication dynamic system as follows:

$$J=\left[\begin{array}{l} \partial F(x)/\partial x\;\partial F(x)/\partial y\;\partial F(x)/\partial z\;\partial F(x)/\partial w\\ \partial F(y)/\partial x\;\partial F(y)/\partial y\;\partial F(y)/\partial z\;\partial F(y)/\partial w\\ \partial F(z)/\partial x\;\partial F(z)/\partial y\;\partial F(z)/\partial z\;\partial F(z)/\partial w\\ \partial F(w)/\partial x\;\partial F(w)/\partial y\;\partial F(w)/\partial z\;\partial F(w)/\partial w \end{array}\right]$$ (33)

### 4.2 Stability condition analysis

According to Lyapunov’s first rule, if the sign of the eigenvalues of the Jacobian matrix is negative, then the equilibrium point is asymptotically stable; If at least one of the eigenvalues of the Jacobian matrix is positive, then this point is unstable. According to Table 2, we can judge that the equilibrium points *E*_1−4_, *E*_7_, and *E*_12−16_ are unstable points. We will discuss this a little here. The stability analysis of the rest of the equilibrium points is as follows:

**Scenario 1**: The stable point (0, 1, 1, 1) means (the government does not subsidize, the automobile enterprises produce new energy vehicles, the banks provide green credit, and the consumers choose to buy new energy vehicles). The stable condition is *R*_*C*1_ > *R*_*C*2_ − *C*_*P*_, *R*_*G*_ + *λ*_1_*CL*_*G*_ + Δ_*BB*_ > *R*_*T*_ − *λ*_2_*CL*_*T*_ − *T*_*B*_ − Δ_*SL*_. At this time, consumers have a higher income from purchasing new energy vehicles, so it is more likely to buy new energy vehicles; The net income of banks choosing green credit strategy is higher than the net income of traditional credit, and banks are more inclined to choose to implement green credit. In this case, automobile enterprises found that the development prospect of new energy vehicles in the market is good through observation. The probability of banks providing green credit is also high, which can alleviate the financial constraints caused by technology research and development and new product production of automobile enterprises to a certain extent. Rational automobile enterprises will be more inclined to produce new energy vehicles. At this time, the government no longer needs too many policies to intervene in the market, and considering that the subsidy policy may increase the pressure of fiscal expenditure, the government will choose to eliminate the subsidy policy, which is a relatively ideal state for the development of any emerging market. Still, the initial stage of industrial development generally requires the government’s policies to regulate the market and finally achieve the ideal natural state.
**Scenario 2**: The stable point (1, 0, 1, 0) means (government subsidies, auto enterprises do not produce new energy vehicles, banks provide green credit, consumers choose not to buy new energy vehicles), and the stable condition is *P*_*A*_ + *S*_1_ < *R*_*ICEV*_ − *F* − *C*_*S*_ − *P*_*B*_, *R*_*G*_ + *λ*_1_*CL*_*G*_ + Δ_*E*_ + *P*_*B*_ > *R*_*T*_ − *λ*_2_*CL*_*T*_ − *T*_*B*_, *R*_*C*2_ − *C*_*P*_ − Δ_*EC*_ > 0. Currently, the income of automobile enterprises producing new energy vehicles is less than that of traditional internal combustion engine vehicles. The income of banks providing green credit is higher than that of formal credit. Consumers can also obtain positive income when purchasing traditional internal combustion engine vehicles. This situation may be in the early stage of developing the new energy vehicle market. At this time, the government provides subsidies through industrial policy support, and the banks also actively provide green credit under the guidance of the national industrial policy. However, due to the early stage of the market development, the development prospects of the new energy vehicle are still being determined for car buyers and sellers, and the production and consumption of traditional internal combustion engine vehicles are still relatively strong. Therefore, in this situation, automobile enterprises will not take risks to produce new energy vehicles, and rational consumers will not try new energy vehicles. Still, this situation will improve with the gradual development of the new energy vehicle market.
**Scenario 3**: The stable point (0, 1, 1, 0) means (the government does not subsidize, the automobile enterprises produce new energy vehicles, the banks provide green credit, and consumers choose not to buy new energy vehicles). The stable condition is *R*_*ICEV*_ − *F* < 0, *R*_*G*_ +*λ*_1_*CL*_*G*_ + Δ_*BB*_ > *R*_*T*_ − *λ*_2_*CL*_*T*_ − *T*_*B*_ − Δ_*SL*_, *R*_*C*1_ < *R*_*C*2_ − *C*_*P*_. Currently, the cost of producing traditional internal-combustion engine vehicles for automobile enterprises rises and may eventually exceed the income. The income of green credit banks is higher than that of traditional credit. The income of consumers purchasing new energy vehicles is lower than that of traditional internal-combustion engine vehicles. Compared with Scenario 2, this situation may be that after the development of the new energy vehicle market for some time, after the adjustment of government policies, the government subsidies will gradually decline until they are finally entirely canceled. The cost of producing traditional internal-combustion engine vehicles for automobile enterprises will rise, resulting in a shrinking income. Automobile enterprises realize the importance of adjusting development strategies and begin to enter the new energy vehicle market in succession. In the case of the decline of government subsidies, banks are still inclined to provide green credit because the benefits of delivering green credit are still more significant than traditional credit. However, at this time, consumers’ willingness to consume new energy vehicles still needs to be stronger. It is necessary to strengthen publicity, guide and improve the public’s environmental awareness, and choose new energy vehicles.
**Scenario 4**: The stable point (0, 1, 0, 1) means (the government does not subsidize, the automobile enterprises produce new energy vehicles, the banks do not provide green credit, and consumers choose to buy new energy vehicles). The stable condition is *R*_*C*1_ > *R*_*C*2_ − *C*_*P*_, *R*_*G*_ + *λ*_1_*CL*_*G*_ + Δ_*BB*_ < *R*_*T*_ − *λ*_2_*CL*_*T*_ − *T*_*B*_ − Δ_*SL*_. Compared with Scenario 3, with the further development of the new energy vehicle market, the technology of automobile enterprises to produce new energy vehicles is more mature. The cost is gradually reduced, the problem of capital constraints on automobile enterprises is gradually weakened, the demand for green capital is also steadily reduced, and the income of green credit provided by banks is also slowly lower than that of traditional credit, so banks will make strategic adjustments, Gradually cancel green credit support. In addition, with the change in consumer consumption concept and the more profound understanding of new energy vehicles, the consumer preference for new energy vehicles will gradually increase, and they will choose progressively new energy vehicles for consumption. This is also a relatively ideal situation. There is a virtuous circle between automobile enterprises and consumers. Automobile enterprises can complete the R&D and production of new energy vehicles without the intervention of foreign funds. Consumers have relatively strong consumption intentions at this stage, and the new energy vehicle market will develop rapidly.
**Scenario 5**: Stable points (1, 0, 0, 0) and (0, 1, 0, 0), with *R*_*ICEV*_ − *F* < 0, *R*_*C*1_ < *R*_*C*2_ − *C*_*P*_, *R*_*G*_ + *λ*_1_*CL*_*G*_ + Δ_*BB*_ < *R*_*T*_ − *λ*_2_*CL*_*T*_ − *T*_*B*_ − Δ_*SL*_, *P*_*A*_ + *S*_1_ < *R*_*ICEV*_ − *F* − *C*_*S*_ − *P*_*B*_, *R*_C2_ − *C*_*P*_ − Δ_*EC*_ > 0, and *R*_*G*_ + *λ*_1_*CL*_*G*_ + Δ_*E*_ + *P*_*B*_ < *R*_*T*_ − *λ*_2_*CL*_*T*_ − *T*_*B*_. The above two situations are not ideal. Whether the government has policy support but the other three parties have not responded, this will further increase the cost of the government to promote the development of the new energy vehicle market, or the automobile enterprises have a solid willingness to produce new energy vehicles. Still, the other three parties have not responded, which will seriously dampen the enthusiasm of the automobile enterprises. Either of these two situations is not beneficial to the development of the whole economy and society and is the most undesirable state. Therefore, the government is required to give subsidies or policy incentives to auto enterprises to produce new energy vehicles and banks to provide green credit to a certain extent, increase the cost of consumers choosing internal combustion engine vehicles and improve their environmental awareness by raising fuel tax and carbon tax, to avoid the stability of the above two conditions and damage the economic and environmental benefits of the whole society.

**Table 2:** Gradual stability analysis of the equilibrium point of the dynamic system of four-party agent replication.

Equilibrium point	Characteristic value *ψ*_1_, *ψ*_2_, *ψ*_3_, *ψ*_4_	Symbol	Stability and conditions
*E*_1_ (0,0,0,0)	*P*_*A*_, *F* − *R*_*ICEV*_, *R*_*G*_ − *R*_*T*_ + *λ*_1_*CL*_*G*_ + *λ*_2_*CL*_*T*_ + *T*_*B*_ + *P*_*B*_, *C*_*P*_ − *R*_*C*2_	+×××	Instable
*E*_2_ (0,0,0,1)	*P*_*A*_, *F* + *R*_*NEV*_, *R*_*G*_ − *R*_*T*_ + *λ*_1_*CL*_*G*_ + *λ*_2_*CL*_*T*_ + *T*_*B*_ + *P*_*B*_, Δ_*BB*_ + Δ*SL*, −(*C*_*P*_ − *R*_*C*2_)	++××	Instable
*E*_3_ (0,0,1,0)	*P*_*A*_, *F* − *R*_*ICEV*_ + *C*_*S*_ + *P*_*B*_, −[*R*_*G*_ − *R*_*T*_ + *λ*_1_*CL*_*G*_ + *λ*_2_*CL*_*T*_ + *T*_*B*_ + *P*_*B*_], *C*_*P*_ − *R*_*C*2_ + Δ_*EC*_	+×××	Instable
*E*_4_ (0,0,1,1)	*P*_*A*_, *F* + *R*_*NEV*_ + *C*_*S*_ + *P*_*B*_ +Δ_*EC*_, −[*R*_*G*_ − *R*_*T*_ + *λ*_1_*CL*_*G*_ + *λ*_2_*CL*_*T*_ + *T*_*B*_ + *P*_*B*_ + Δ_*BB*_ + Δ_*SL*_], −(*C*_*P*_ − *R*_*C*2_ + Δ_*EC*_)	++××	Instable
*E*_5_ (0,1,0,0)	−*S*_1_, −(*F* − *R*_*ICEV*_), *R*_*G*_ − *R*_*T*_ + *λ*_1_*CL*_*G*_ + *λ*_2_*CL*_*T*_ + *T*_*B*_, *C*_*P*_ − *R*_*C*2_ + *R*_*C*1_	-×××	Stable when conditions 6, 7 and 8 are met
*E*_6_ (1,0,0,0)	−*P*_*A*_, *F* − *R*_*ICEV*_ + *P*_*A*_ + *S*_1_, *R*_*G*_ − *R*_*T*_ + *λ*_1_*CL*_*G*_ + *λ*_2_*CL*_*T*_ + *T*_*B*_ + *P*_*B*_ + Δ_*E*_, *C*_*P*_ − *R*_*C*2_	-×××	Stable when conditions 3, 5 and 9 are met
*E*_7_ (1,0,0,1)	−*P*_*A*_, *F* + *P*_*A*_ + *S*_1_ + *R*_*NEV*_, *R*_*G*_ − *R*_*T*_ + *λ*_1_*CL*_*G*_ + *λ*_2_*CL*_*T*_ + *T*_*B*_ + *P*_*B*_ + Δ_*BB*_ + Δ_*SL*_ + Δ_*E*_, −(*C*_*P*_ − *R*_*C*2_)	-+××	Instable
*E*_8_ (0,1,0,1)	−*S*_1_ − *S*_2_, −(*F* + *R*_*NEV*_), *R*_*G*_ − *R*_*T*_ + *λ*_1_*CL*_*G*_ + *λ*_2_*CL*_*T*_ + *T*_*B*_ + Δ_*BB*_ + Δ_*SL*_, −(*C*_*P*_ − *R*_*C*2_ + *R*_*C*1_)	--××	Stable when conditions 1 and 8 are met
*E*_9_ (0,1,1,0)	−*S*_1_ − *λ*_1_*CL*_*G*_, −(*F* − *R*_*ICEV*_ + *P*_*B*_ + *C*_*S*_), −[*R*_*G*_ − *R*_*T*_ + *λ*_1_*CL*_*G*_ + *λ*_2_*CL*_*T*_ + *T*_*B*_ + Δ_*BB*_ + Δ_*SL*_], *C*_*P*_ − *R*_*C*2_ + *R*_*C*1_	-×××	Stable when conditions 2, 6 and 7 are met
*E*_10_ (1,0,1,0)	−*P*_*A*_, *F* − *R*_*ICEV*_ + *P*_*A*_ + *S*_1_ + *C*_*S*_ + *P*_*B*_, −[*R*_*G*_ − *R*_*T*_ + *λ*_1_*CL*_*G*_ + *λ*_2_*CL*_*T*_ + *T*_*B*_ + *P*_*B*_ + Δ_*E*_], *C*_*P*_ − *R*_*C*2_ + Δ_*EC*_	-×××	Stable when conditions 3, 4 and 5 are met
*E*_11_ (0,1,1,1)	−*S*_1_ −*λ*_1_*CL*_*G*_ − *S*_2_, −(*F* + *R*_*NEV*_ + *P*_*B*_ + *C*_*S*_ + Δ_*EC*_), −[*R*_*G*_ − *R*_*T*_ + *λ*_1_*CL*_*G*_ + *λ*_2_*CL*_*T*_ + *T*_*B*_ + Δ_*SL*_ + Δ_*BB*_], −(*C*_*P*_ − *R*_*C*2_ + *R*_*C*1_)	--××	Stable when conditions 1 and 2 are met
*E*_12_ (1,0,1,1)	−*P*_*A*_, *F* + *C*_*S*_ + *P*_*B*_ + *P*_*A*_ + *S*_1_ + *R*_*NEV*_ + Δ_*EC*_, −[*R*_*G*_ − *R*_*T*_ + *λ*_1_*CL*_*G*_ + *λ*_2_*CL*_*T*_ + *T*_*B*_ + Δ_*E*_ + *P*_*B*_ + Δ_*SL*_ + Δ_*BB*_], −(*C*_*P*_ − *R*_*C*2_ + Δ_*EC*_)	-+××	Instable
*E*_13_ (1,1,0,1)	*S*_1_ + *S*_2_, −(*F* + *P*_*A*_ + *S*_1_ + *R*_*NEV*_), *R*_*G*_ − *R*_*T*_ + *λ*_1_*CL*_*G*_ + *λ*_2_*CL*_*T*_ + *T*_*B*_ + Δ_*E*_ + Δ_*SL*_ + Δ_*BB*_, −(*C*_*P*_ − *R*_*C*2_ + *R*_*C*1_ + *S*_2_)	+-××	Instable
*E*_14_ (1,1,0,0)	*S*_1_, −(*F* − *R*_*ICEV*_ + *P*_*A*_ + *S*_1_), −[*R*_*G*_ − *R*_*T*_ + *λ*_1_*CL*_*G*_ + *λ*_2_*CL*_*T*_ + *T*_*B*_ + Δ_*E*_], *C*_*P*_ − *R*_*C*2_ + *R*_*C*1_ + *S*_2_	+-××	Instable
*E*_15_ (1,1,1,0)	*S*_1_ + *λ*_1_*CL*_*G*_, −(*F* − *R*_*ICEV*_ + *P*_*B*_ + *C*_*S*_ + *P*_*A*_ + *S*_1_), −[*R*_*G*_ − *R*_*T*_ + *λ*_1_*CL*_*G*_ + *λ*_2_*CL*_*T*_ + *T*_*B*_ + Δ_*E*_], *C*_*P*_ − *R*_*C*2_ + *R*_*C*1_ + *S*_2_	+×××	Instable
*E*_16_ (1,1,1,1)	*S*_1_ + *λ*_1_*CL*_*G*_ + S_2_, −(*F* + *P*_*B*_ + *C*_*S*_ + *P*_*A*_ + *S*_1_ + *R*_*NEV*_ + Δ_*EC*_) −[*R*_*G*_ − *R*_*T*_ + *λ*_1_*CL*_*G*_ + *λ*_2_*CL*_*T*_ + *T*_*B*_ + Δ_*E*_ + Δ_*BB*_ + Δ_*SL*_], −(*C*_*P*_ − *R*_*C*2_ + *R*_*C*1_ + *S*_2_)	+-××	Instable

Note: × represents positive and negative uncertainty, the same below. Condition 1: *R*_*C*1_ > *R*_*C*2_ − *C*_*P*_; Condition 2: *R*_*G*_ + *λ*_1_*CL*_*G*_ + Δ_*BB*_ > *R*_*T*_ − *λ*_2_*CL*_*T*_ − *T*_*B*_ − Δ_*SL*_;Condition 3: *P*_*A*_ + *S*_1_ < *R*_*ICEV*_ − *F* − *C*_*S*_ − *P*_*B*_; Condition 4: *R*_*G*_ + *λ*_1_*CL*_*G*_ + Δ_*E*_ + *P*_*B*_ > *R*_*T*_ − *λ*_2_*CL*_*T*_ − *T*_*B*_; Condition 5: *R*_*C*2_ − *C*_*P*_ − Δ_*EC*_ > 0;Condition 6: *R*_*ICEV*_ − *F* < 0; Condition 7: *R*_*C*1_ < *R*_*C*2_ − *C*_*P*_; Condition 8: *R*_*G*_ + *λ*_1_*CL*_*G*_ + Δ_*BB*_ < *R*_*T*_ − *λ*_2_*CL*_*T*_ − *T*_*B*_ − Δ_*SL*_; Condition 9: *R*_*G*_ + *λ*_1_*CL*_*G*_ + Δ_*E*_ + *P*_*B*_ < *R*_*T*_ − *λ*_2_*CL*_*T*_ − *T*_*B*_.

## 5 Numerical simulation analysis

To more intuitively describe the behavior and strategy choice of multiple participants in the development process of the new energy vehicle industry, the next step is to use the MATLAB tool to conduct numerical simulation analysis, focusing on the evolution of the strategy choice of the government, automobile enterprises, banks, and consumers.

### 5.1 Numerical simulation analysis under ideal strategy combination

**Scenario 1**: According to the estimation of the actual situation, condition (1) and condition (2) are satisfied by the parameter assignment [27, 37]. The assignment of parameters in the evolutionary game system is as follows: *P*_*A*_ = 2, *S*_1_ = 9, *λ*_1_*CL*_*G*_ = 3, *S*_2_ = 7, *F* = 2, *C*_*S*_ = 1, *P*_*B*_ = 1, *R*_*ICEV*_ = 6, *R*_*NEV*_ = 5, Δ_*EC*_ = 0.5, Δ_*SL*_ = 0.5, Δ_*BB*_ = 0.5, Δ_*E*_ = 1, *λ*_2_*CL*_*T*_ = 3, *R*_*G*_ = 8, *R*_*T*_ = 15, *T*_*B*_ = 2, *C*_*P*_ = 2, *R*_*C*2_ = 5, *R*_*C*1_ = 4.

(1) The impact of subsidies and changes in consumers’ income from purchasing new energy vehicles on the strategic choices of all parties

**Fig 6:**
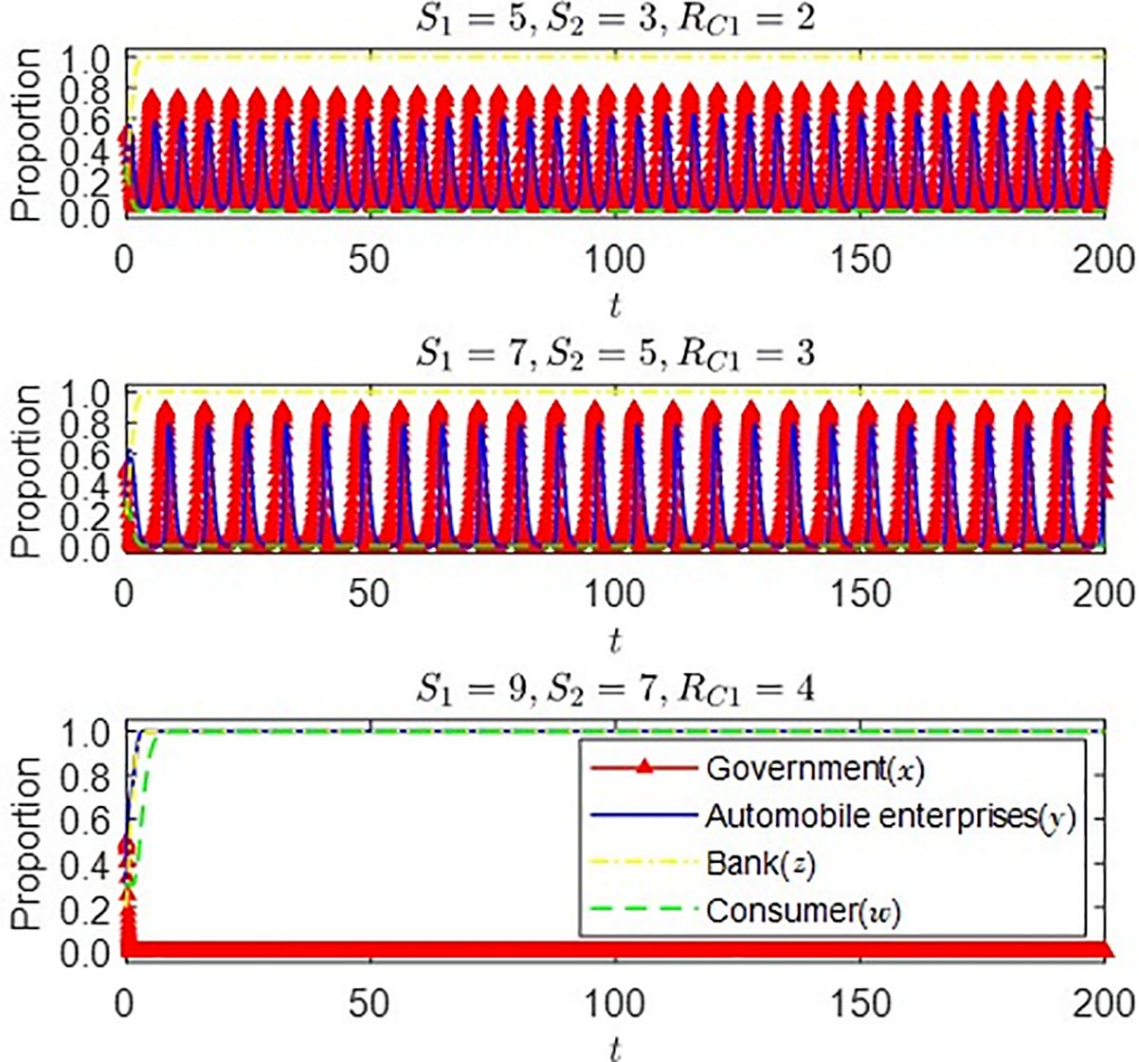
Impact of *S*_1_, *S*_2_, and *R*_*C*1_ changes on the evolution of each agent’s strategy under the conditions of (1) and (2).

Based on the above assignment, through the changes of *S*_1_, *S*_2_, and *R*_*C*1_, we can get Fig 6:

It can be seen from Fig 6 that with the gradual increase of consumers’ income (*R*_*C*1_) and government subsidies (*S*_1_, *S*_*2*_) from purchasing new energy vehicles, consumers’ strategic choices will gradually shift from the strategy of not buying new energy vehicles to the strategy of purchasing new energy vehicles. Automobile enterprises’ strategic choices fluctuate with the gradual change of government subsidies. The greater the subsidies, the higher the incentive level for auto companies. The strategic decisions of auto companies will also be stable in producing new energy vehicles. As the government gradually reduces its subsidies and finally stabilizes in the de-subsidy strategy, the market will operate more smoothly at this time, and its contribution to the social economy will gradually increase.

(2) The impact of bank green credit income and enterprise cost increase on the strategic choice of all parties

**Fig 7:**
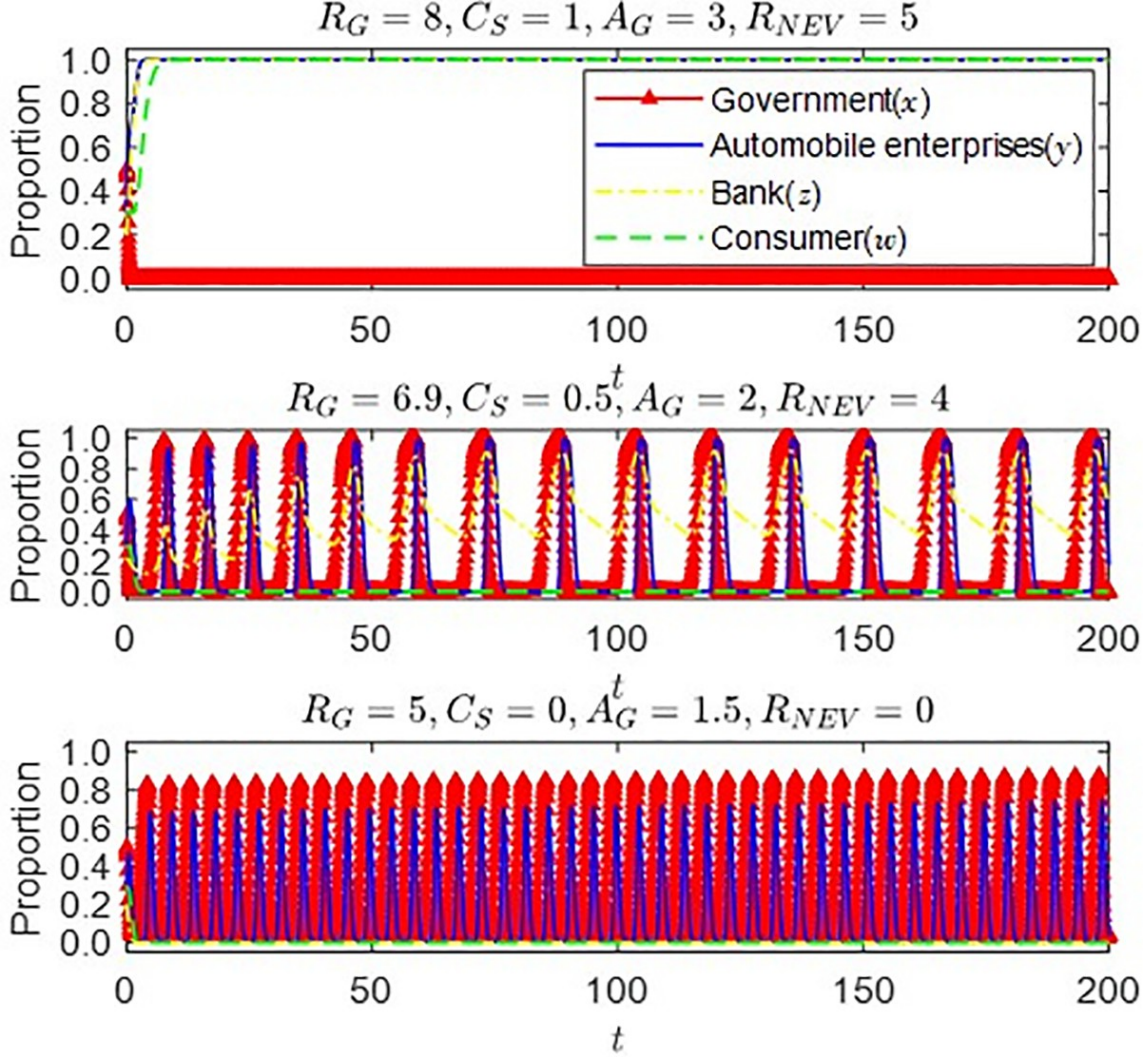
Impact of changes in *R*_*G*_, *C*_*S*_, *A*_*G*_ and *R*_*NEV*_ on strategy evolution of all parties.

Similarly, under the condition that conditions (1) and (2) are initially satisfied, we can obtain Fig 7 by changing *R*_*G*_, *C*_*S*_, and *λ*_1_*CL*_*G*_ (represented by *A*_*G*_ in Fig 2) and *R*_*NEV*_:

It can be seen from Fig 7 that with the government’s incentives for banks to provide green credit and the banks’ income from providing green credit decreasing gradually, the banks’ strategic choices change to implement green credit strategy—hover between the two strategies—choose traditional credit strategy instead of providing green credit strategy. With the gradual shrinkage of green credit in the capital market, the government has gradually adopted the subsidy strategy to ensure the stable development of the new energy vehicle market. Still, the strategy choice at this time is unstable, and the strategic choice of automobile enterprises could be more stable. When the bank strategy wanders, the traditional credit strategy is selected instead of providing a green credit strategy. There is a gap between the auto enterprises and the government at the peak of strategy selection. The cancellation of the green credit policy has further exacerbated the problem of capital constraints on auto enterprises, reducing the probability of auto enterprises carrying out technology research and development and eventually producing new energy vehicles. Green credit plays a role in developing new energy vehicles to a certain extent, significantly when government subsidies fluctuate. Therefore, the government should encourage banks to provide green credit while implementing the subsidy policy to solve the financial constraints of new technology research and development of enterprises, significantly when subsidies decline yearly, to ensure the stable growth of China’s new energy vehicle market.

(3) The impact of different initial states of banks on the strategic choice of the parties

**Fig 8:**
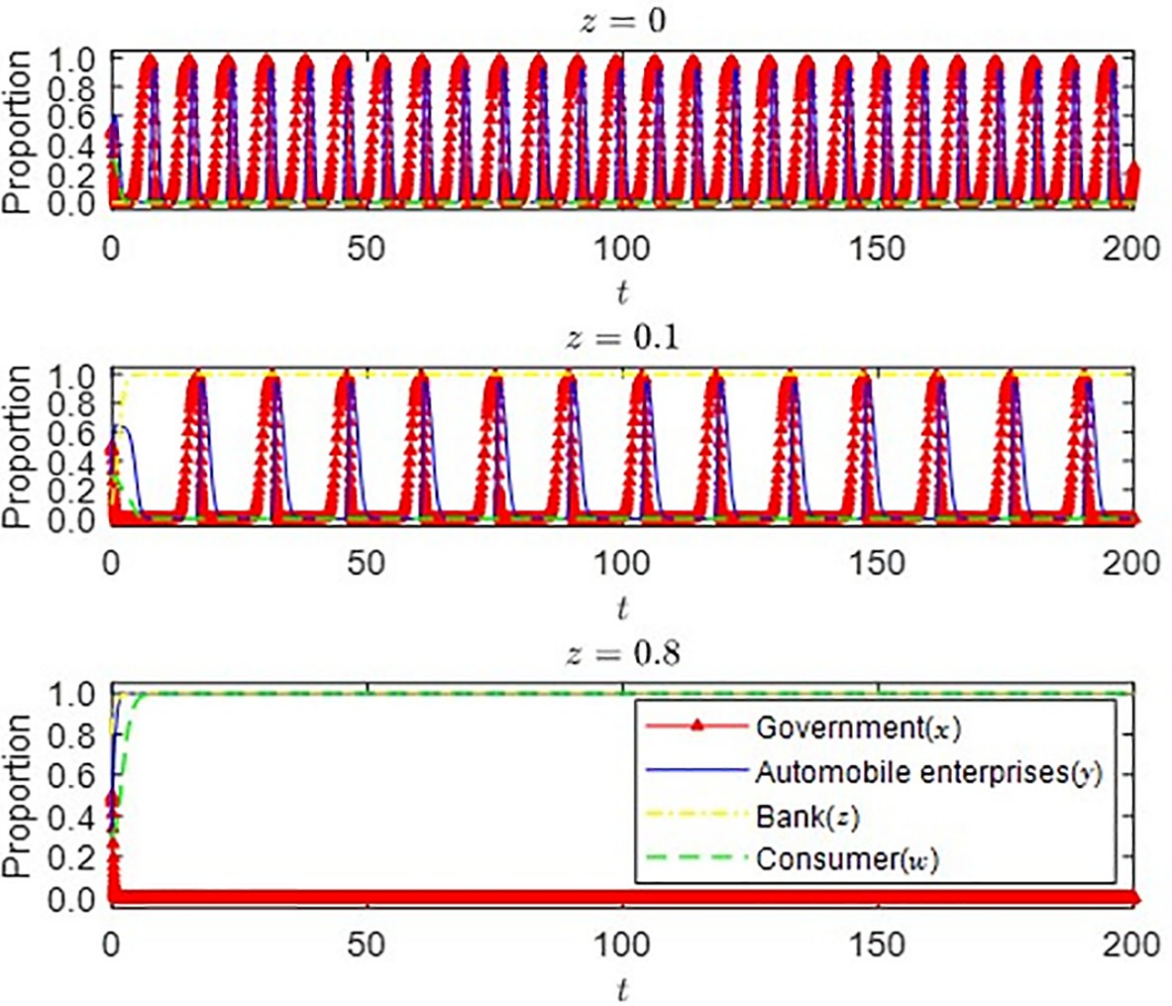
Strategy selection of all parties under different initial states of banks.

It can be seen from Fig 8 that when the initial proportion of the strategic choice of banking institutions changes from *z* = 0 to 0.1, because the market development situation is still unclear, auto companies still rely more on the government’s subsidy policy. In this case, consumers remain wait-and-see and must be determined to buy new energy vehicles. The bank’s green credit policy does not play a supplementary role in adjusting the government’s subsidy policy. However, when the initial proportion rises to 0.8, it can be seen from the figure that the strategic choice of the bank is to provide green credit rapidly and steadily, and auto enterprises and consumers also choose to produce and purchase new energy vehicles. The government no longer needs to offer many financial subsidies to stimulate market development. It only needs to give full play to the complementary role of green credit to financial grants to maintain the high-quality development of the new energy vehicle market. Currently, the bank’s green credit policy has a more apparent supplementary effect on adjusting the subsidy policy.

**Scenario 2**: According to the estimation of the actual situation, conditions (3), (4), and (5) are satisfied by parameter assignment. The assignment of parameters in the evolutionary game system is as follows: *P*_*A*_ = 2, *S*_1_ = 9, *λ*_1_*CL*_*G*_ = 3, *S*_2_ = 7, *F* = 2, *C*_*S*_ = 1, *P*_*B*_ = 1, *R*_*ICEV*_ = 16, *R*_*NEV*_ = 5, Δ_*EC*_ = 0.5, Δ_*SL*_ = 0.5, Δ_*BB*_ = 0.5, Δ_*E*_ = 1, *λ*_2_*CL*_*T*_ = 3, *R*_*G*_ = 8, *R*_*T*_ = 15, *T*_*B*_ = 2, *C*_*P*_ = 2, *R*_*C*2_ = 5, *R*_*C*1_ = 4.

(1) The impact of the change of the traditional internal combustion engine vehicle revenue of the automobile enterprises on the strategic choice of the main parties

**Fig 9:**
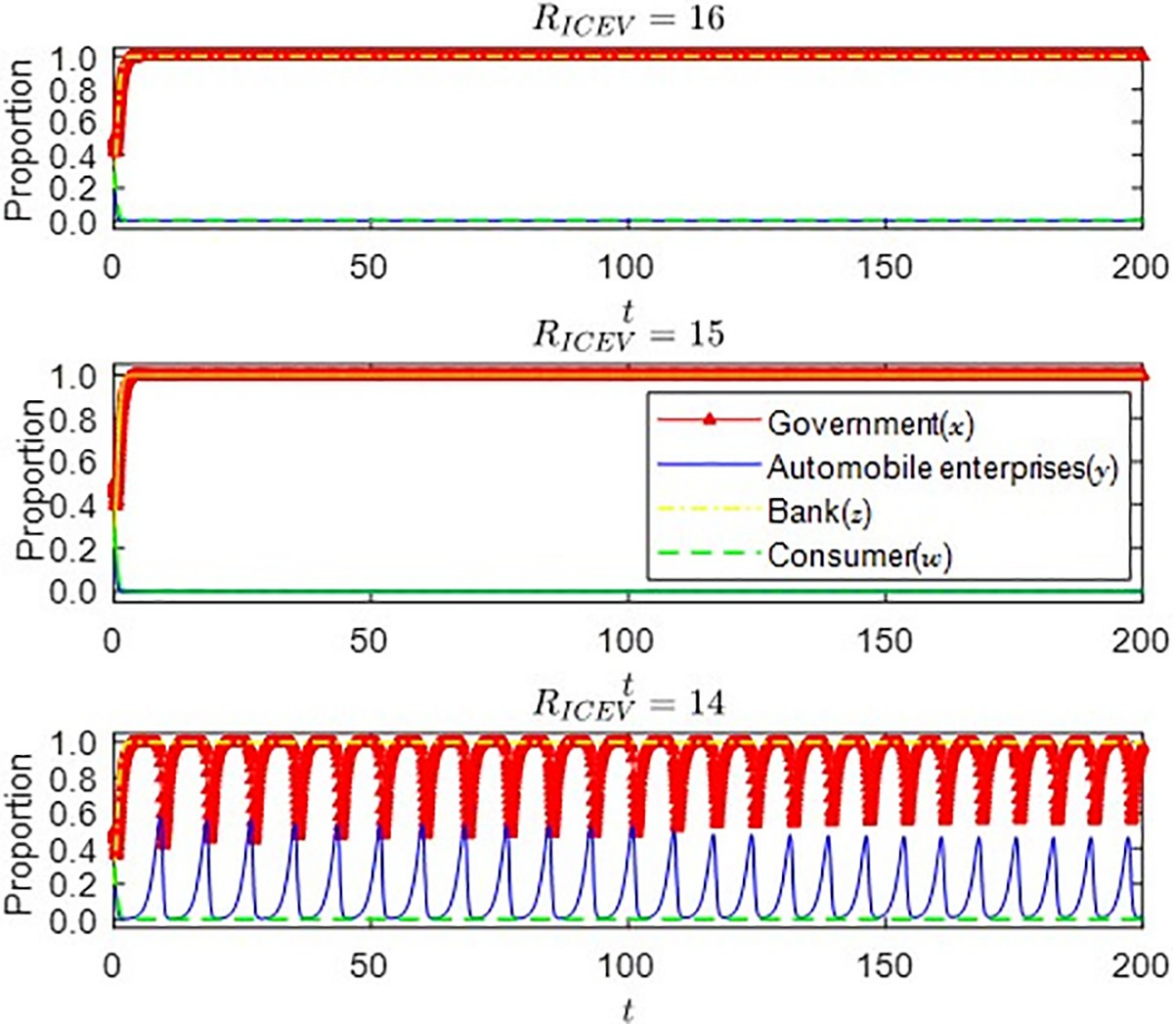
Impact of income changes brought by traditional internal combustion engine vehicles.

It can be seen from Fig 9 that the strategic choice of automobile enterprises is mainly affected by the benefits that the traditional internal combustion engine vehicles and new energy vehicles can bring to the enterprise, which also conforms to the assumption of the principle of enterprise profit maximization. Secondly, when *R*_*ICEV*_ = 15, it is at the boundary point where the two types of vehicles benefit the enterprise. Currently, the two types of vehicles bring the same benefits to the enterprise. Still, the automobile enterprises are relatively mature, and the market is relatively stable due to the production technology of traditional internal combustion engine vehicles. Therefore, even with the support of government subsidies, enterprises still prefer the production of traditional internal combustion engines. When *R*_*ICEV*_ = 14, at this time, it may be affected by the national environmental regulation policy. The income of traditional internal combustion engine vehicles gradually starts to be lower than that of new energy vehicles. At the same time, under the condition of government subsidies, one after another, automobile enterprises begin to gradually change their production strategies and start to produce new energy vehicles.

(2) The impact of government subsidy changes on the strategic choices of various parties under the revenue boundary point of automobile enterprises

**Fig 10:**
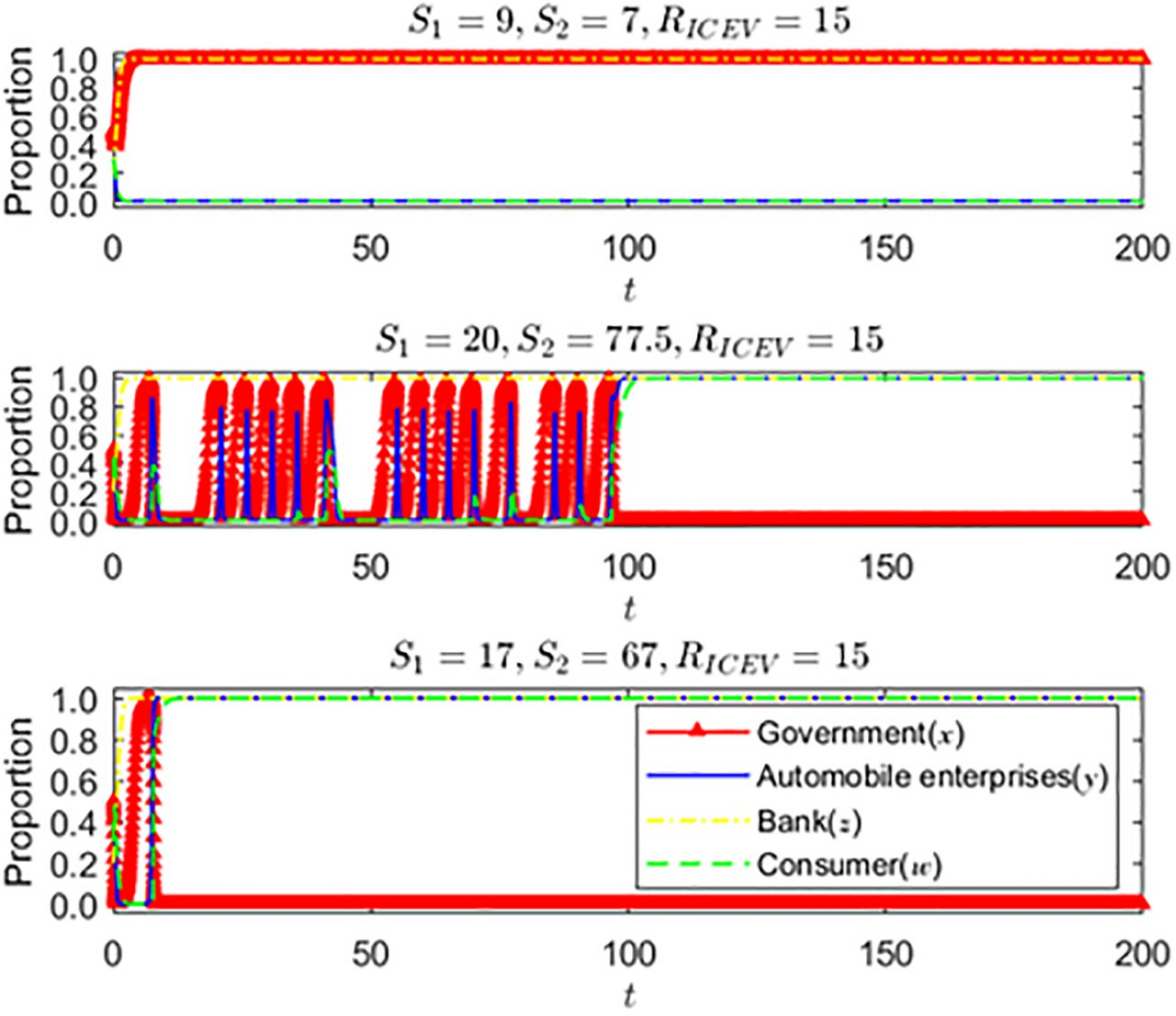
Consider the impact of changes in government subsidies on the strategic choices of consumers and auto companies.

It can be seen from Fig 10 that when automobile enterprises produce two types of cars, the benefits are equal. That is, when they are at the boundary point, the impact of the change of government subsidy strategy on the automobile enterprises and consumers’ strategic choices is investigated. With the increase in government subsidies, the strategic choices of consumers and auto companies begin to change. Eventually, when the government subsidies reach a certain level, they will be stable in the strategic choices of auto companies to produce and consumers to buy new energy vehicles. At this time, government subsidies will gradually decrease. It can be seen from the figure that when the government subsidies are slightly reduced, the speed at which automobile enterprises and consumers can stabilize their production and purchase strategies changes. It can be inferred that at this time, the government can gradually adjust the subsidy strategy while alleviating the financial pressure on the premise of ensuring the regular operation of the new energy vehicle market until it is finally canceled, which is also consistent with the trend of the national subsidy policy for the new energy vehicle market.

(3) The impact of different initial states of consumers on the strategic choice of the main parties

**Fig 11:**
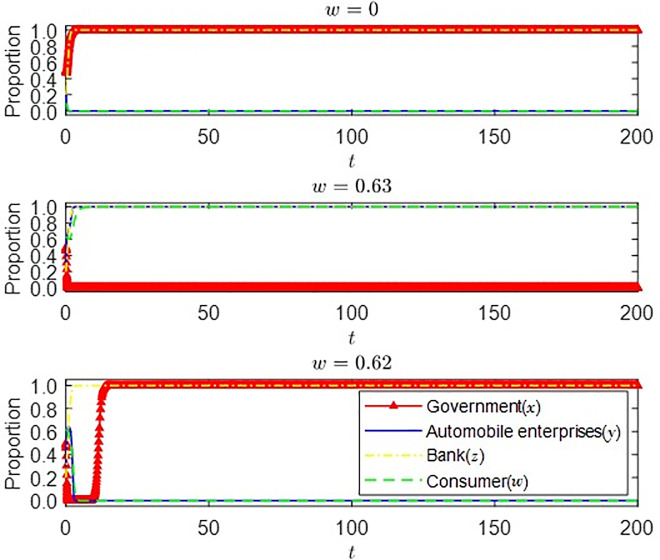
Strategy choices of consumers in different initial states.

It can be seen from Fig 11 that when the initial proportion of consumers’ strategic choices changes from 0 to 0.63, the government’s strategic choice changes from subsidy to non-subsidy, and the strategic choice of automobile enterprises changes from non-production to production of new energy vehicles. With the increase of the initial proportion of consumers, their willingness to buy new energy vehicles also gradually increases. At this time, automobile enterprises will progressively change their production strategies when they catch business opportunities. With the steady development of the new energy vehicle market, the government’s subsidy strategy will be adjusted and eventually withdrawn from the new energy vehicle market, which is consistent with the actual situation in China. When the initial proportion of consumer strategy choice is reduced from 0.63 to 0.62, the strategic choice of auto companies is changed to not producing new energy vehicles. The government finally chose a subsidy strategy after experiencing short-term fluctuations. It can be inferred that there is a threshold value for the initial proportion of consumer strategy choice, which is between *w* = 0.62 and *w* = 0.63. After reaching the threshold value, the probability of consumers finally buying new energy vehicles is high. Good market prospects will affect auto companies to change their production strategies. Still, consumers will unlikely buy new energy vehicles when it is lower than the threshold. At this time, auto companies are not optimistic about the development prospects of the new energy vehicle market. In this case, to promote the development of the new energy vehicle market, the government must stimulate the development of emerging markets through subsidies, tax cuts, and other ways.

### 5.2 Analysis of the impact of government subsidy mechanism on the strategic choice of all parties

**Fig 12:**
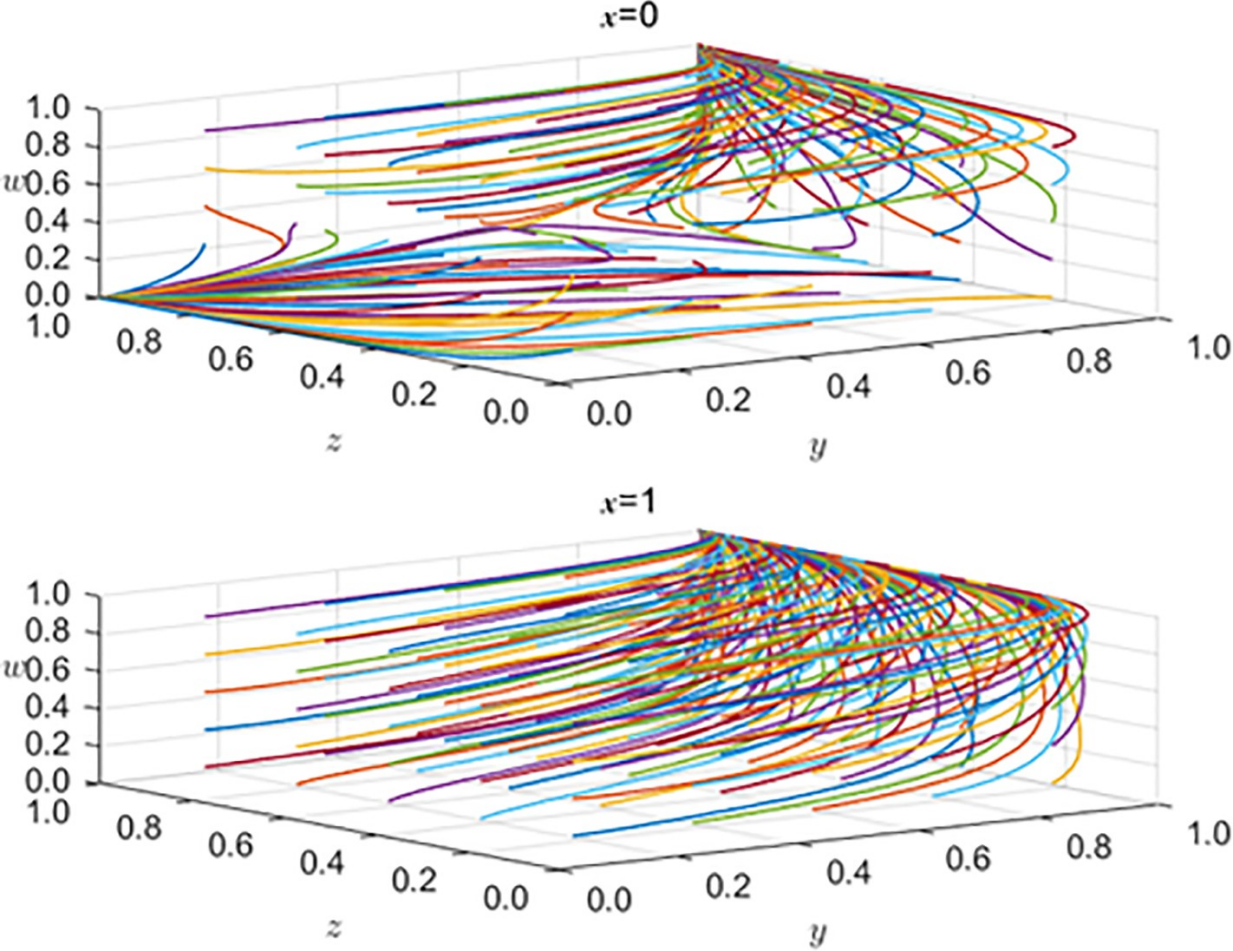
The impact of government subsidy strategy on the strategic choices of all parties.

To further verify the effectiveness of the influence of the government subsidy mechanism on the development strategy selection of the new energy automobile industry, we set *x* = 0 and *x* = 1 to represent the two strategic choices of government subsidies and subsidies to observe the evolution process of the other three main strategic choices. The simulation results are shown in Fig 12.

As can be seen from Fig 12, when *x* = 0, that is, the government does not choose a subsidy strategy, there is no unique stable strategy for the strategic choice of each entity in the new energy vehicle market, that is, at this time, each entity is on the sidelines. There is significant uncertainty about the emerging market. Due to too many uncertain factors, it is difficult for each entity to form a consistent, stable strategy in the spontaneous strategic choice. Therefore, government macro-control is needed to promote the development of emerging markets. When *x* = 1 means that the government implements subsidy policies, the strategic choices of various entities gradually tend to evolve to the only stable point, namely, stable strategies for automobile enterprises to produce new energy vehicles, banks to provide green credit, and consumers to purchase new energy vehicles. This indicates the critical impact of government macro-control on the development of the entire new energy vehicle market, further verifying the effectiveness of the government subsidy mechanism.

Based on the above, when the government does not provide subsidies and when banks provide green credit, there are stable strategies for some automotive companies and consumers to choose to produce and purchase new energy vehicles, as well as stable strategies for other automotive companies and consumers to choose not to produce and not to buy. In the absence of government subsidies, green credit can still play a role in supporting the development of the green economy, stimulating and guiding the behavior choices of automobile enterprises and consumers. However, relying solely on the support of green credit policies can only partially mobilize the production and consumption behavior of automobile enterprises and consumers, which also verifies the complementary role of green credit policies in the decline of government subsidies to a certain extent. In addition, when the government implements the subsidy strategy, the strategic choices of the four parties tend to be stable, reflecting the fact that government subsidies and green credit need to cooperate to maximize the effectiveness of relevant incentive policies better. The development of the new energy vehicle industry is ultimately bound to be market-oriented, and the exit of state subsidies is an inevitable trend. At this time, the green credit policy’s complementary role in developing the new energy vehicle market will be further strengthened, and its role will become even more critical.

## 6 Research conclusions and policy implications

This study is based on the background of high-quality national development, considering changes in government subsidies, bank green credit, and consumer participation in the development strategy selection of the new energy vehicle industry. It draws the following conclusions and policy implications.

Based on the above research, this article draws the following conclusions:

First, the government’s subsidy mechanism significantly promotes the development of the new energy vehicle industry, including subsidies for automotive companies and the reduction and exemption of consumer purchase taxes. However, the government’s subsidy policy has a threshold value beyond which its role in promoting market development will weaken. At the same time, it may also bring significant financial pressure on the government.

Secondly, in the context of the gradual decline in national subsidies, bank green credit is an essential supplement to national policy adjustments. Solving the financial constraints automotive companies face in developing and producing new energy vehicles is crucial. The bank’s green credit policy is mainly affected by factors such as the net income brought by green credit itself, national policy incentives, and risk control.

Thirdly, in the context of the country’s green and low-carbon development, enterprises, as market entities pursuing maximum benefits, shoulder the dual responsibility of protecting the environment and promoting economic growth. Therefore, the green production behavior of enterprises is crucial for the green and high-quality development of the national economy. Whether an automobile enterprise produces new energy vehicles mainly depends on factors such as its net income relative to traditional internal combustion engine vehicles, government policy support, the punishment for "fraudulent compensation" and "loan fraud", and the market sales prospects of new energy vehicles.

Finally, cultivating green concepts and promoting green consumption by consumers is an inherent requirement for promoting high-quality economic development and is significant for achieving the dual carbon goal. As rational economic people in the consumption market of new energy vehicles, consumers’ strategic choices based on maximizing their utility are mainly influenced by the utility that new energy vehicle consumption can bring and the benefits and costs of traditional internal combustion engine vehicles.

Based on the above conclusions, this article draws the following policy implications:

1. The government should always pay attention to the development and changes of various stakeholders in the market during the process of implementing macroeconomic control policies and should control the implementation of policies. In the early stages of the development of emerging markets, it should focus on pulling, at which time the appropriate policy incentive effect may be more prominent. When the industry enters a stable period of development, it is necessary to pay attention to appropriate power reduction and reduce the excessive dependence of market entities on fiscal policies to ensure the healthy development of the market and promote high-quality economic development, which is also in line with China’s dynamic policy adjustment for the new energy vehicle industry.
2. The government should actively guide banking institutions to participate in the green financial market to fully play the complementary role of green credit for macro government policies. In addition, while actively assuming social responsibility, banks will pay more attention to the issue of maximizing their interests, which also requires ensuring incentives for banks’ green credit to promote the construction of a diversified system of green financial products for banks so that they can fully play their role in the financial market. At the same time, optimize the development environment of the green financial market to effectively address the information asymmetry and moral hazard issues between banks and enterprises and ultimately reduce the uncertainty risks that banks may face in carrying out green financial services to ensure its role and ultimately promote the development of green industries.
3. The country must attach importance to policy guidance for emerging markets, increase the cost of using internal combustion engine vehicles by raising the carbon tax and fuel cost of traditional internal combustion engine vehicles, and increase the benefits of new energy vehicles can bring through subsidies. In addition, it is necessary to actively promote the establishment of strategic goals for the development of clean energy, promote low-carbon development of enterprises, increase the punishment for "fraudulent compensation" and "fraudulent loan" behavior of automobile enterprises, improve the environmental awareness and social responsibility of automobile production enterprises, and urge them to produce new energy vehicles. On this basis, it is also necessary to actively stimulate consumers’ green consumption behavior to balance supply and demand in the new energy vehicle market and maintain a stable and healthy market development.
4. It is required to provide appropriate subsidies or reduce relevant taxes for consumers’ green consumption behavior to improve the effectiveness of purchasing new energy vehicles. Increasing the use costs of traditional internal combustion engine vehicles, such as fuel tax and purchase tax, can reduce the point of internal combustion engine vehicle consumption. In addition, it is also possible to enhance consumers’ green concepts through national environmental publicity, guide consumers to purchase new energy vehicles, and provide significant momentum for developing the new energy vehicle market.

## Supporting information

S1 File Numerical simulation program and parameter assignment. (PDF)
