# Supplementary material for: Research on the development strategy selection of the new energy vehicle industry from the perspective of green credit—Based on the foursquare evolutionary game analysis
Source: PLoS One. 2024 Jan 29;19(1):e0297813. doi: 10.1371/journal.pone.0297813 (PMC10824455; doi:10.1371/journal.pone.0297813)
Supplement: S1 File — (PDF) [file pone.0297813.s001.pdf]

```
function dydt=sifang(t,y,PA,S1,L1CLG,S2,F,CS,PB,RI,RN,EC,SL,BB,E,L2CLT,RG,RT,TB,CP,RC2,RC1)
dydt=zeros(4,1);
dydt(1)=-y(1)*(y(1) - 1)*(PA -y(2)*(PA+S1+y(3)*L1CLG+y(4)*S2));
dydt(2)=-y(2)*(y(2) - 1)*(FRI+y(3)*(CS+PB)+y(1)*(PA+S1)+y(4)*(RI+RN+y(3)*EC));
dydt(3)=-y(3)*(y(3)-1)*((1-y(2))*PB+y(4)*(SL+BB)+y(1)*E+L1CLG+L2CLT+RG-RT+TB);
dydt(4)=-y(4)*(y(4) - 1)*(CP-RC2+(1-y(2))*y(3)*EC+y(2)*RC1+y(1)*y(2)*S2);
end
```

**Figure(6)**

```
clc;clear;
PA=2,S1=5,L1CLG=3,S2=3,F=2,CS=1,PB=1,RI=6,RN=5,EC=0.5,SL=0.5,BB=0.5,E=1,L2CLT=3,RG=8,RT=15,TB=2,CP=2,RC2=5,RC1=2;
%subplot(3,1,1)
set(0,'defaultfigurecolor','w')
[t,y]=ode45(@(t,y) sifang(t,y,PA,S1,L1CLG,S2,F,CS,PB,RI,RN,EC,SL,BB,E,L2CLT,RG,RT,TB,CP,RC2,RC1),[0,200],[0.4,0.3,0.2,0.3]);
points=1:1:length(t);
plot(t,y(:,1),'r^-','linewidth',1,'markersize',3,'markerfacecolor','r','markerindices',points);
hold on
plot(t,y(:,2),'b-','linewidth',1);
hold on
plot(t,y(:,3),'y-.','linewidth',1);
hold on
plot(t,y(:,4),'g--','linewidth',1);
hold on
set(gca,'XTick',[0:50:200],'YTick',[0.0:0.2:1.0])
set(gca,'YTickLabel',num2str(get(gca,'YTick')','%.1f'));
axis([0 200 -0.05 1.05])
xlabel('$t$','interpreter','latex');
ylabel('Proportion');
zhuti=title('$S_{1}=5, S_{2}=3,R_{C1}=2$');
set(zhuti,'interpreter','latex')
legend('Government({\it\fontname{Bodoni MT}x})','Automobile enterprises ({\it\fontname{Bodoni MT}y})','Bank({\it\fontname{Bodoni MT}z})','Consumer({\it\fontname{Bodoni MT}w})');
PA=2,S1=7,L1CLG=3,S2=5,F=2,CS=1,PB=1,RI=6,RN=5,EC=0.5,SL=0.5,BB=0.5,E=1,L2CLT=3,RG=8,RT=15,TB=2,CP=2,RC2=5,RC1=3;
%subplot(3,1,1)
set(0,'defaultfigurecolor','w')
[t,y]=ode45(@(t,y) sifang(t,y,PA,S1,L1CLG,S2,F,CS,PB,RI,RN,EC,SL,BB,E,L2CLT,RG,RT,TB,CP,RC2,RC1),[0,200],[0.4,0.3,0.2,0.3]);
points=1:1:length(t);
```

```
plot(t,y(:,1),'r^-','linewidth',1,'markersize',3,'markerfacecolor','r','markerindices',points);
hold on
plot(t,y(:,2),'b-','linewidth',1);
hold on
plot(t,y(:,3),'y-.','linewidth',1);
hold on
plot(t,y(:,4),'g--','linewidth',1);
hold on
set(gca,'XTick',[0:50:200],'YTick',[0.0:0.2:1.0])
set(gca,'YTickLabel',num2str(get(gca,'YTick')','%.1f'));
axis([0 200 -0.05 1.05])
xlabel('$t$','interpreter','latex');
ylabel('Proportion');
zhuti=title('$S_{1}=5, S_{2}=3,R_{C1}=2$');
set(zhuti,'interpreter','latex')
legend('Government({\it\fontname{Bodoni MT}x})','Automobile enterprises
({\it\fontname{Bodoni MT}y})','Bank({\it\fontname{Bodoni
MT}z})','Consumer({\it\fontname{Bodoni MT}w})');
PA=2,S1=9,L1CLG=3,S2=7,F=2,CS=1,PB=1,RI=6,RN=5,EC=0.5,SL=0.5,BB=0.5,E=1,
L2CLT=3,RG=8,RT=15,TB=2,CP=2,RC2=5,RC1=4;
%subplot(3,1,1)
set(0,'defaultfigurecolor','w')
[t,y]=ode45(@(t,y)
sifang(t,y,PA,S1,L1CLG,S2,F,CS,PB,RI,RN,EC,SL,BB,E,L2CLT,RG,RT,TB,CP,RC2,R
C1),[0,200],[0.4,0.3,0.2,0.3]);
points=1:1:length(t);
plot(t,y(:,1),'r^-','linewidth',1,'markersize',3,'markerfacecolor','r','markerindices',points);
hold on
plot(t,y(:,2),'b-','linewidth',1);
hold on
plot(t,y(:,3),'y-.','linewidth',1);
hold on
plot(t,y(:,4),'g--','linewidth',1);
hold on
set(gca,'XTick',[0:50:200],'YTick',[0.0:0.2:1.0])
set(gca,'YTickLabel',num2str(get(gca,'YTick')','%.1f'));
axis([0 200 -0.05 1.05])
xlabel('$t$','interpreter','latex');
ylabel('Proportion');
zhuti=title('$S_{1}=5, S_{2}=3,R_{C1}=2$');
set(zhuti,'interpreter','latex')
legend('Government({\it\fontname{Bodoni MT}x})','Automobile enterprises
({\it\fontname{Bodoni MT}y})','Bank({\it\fontname{Bodoni
MT}z})','Consumer({\it\fontname{Bodoni MT}w})');
```

## Figure(12)

```
clc;clear;
```

```
PA=2,S1=9,L1CLG=3,S2=7,F=2,CS=1,PB=1,RI=6,RN=5,EC=0.5,SL=0.5,BB=0.5,E=1,L2CLT=3,RG
=8,RT=15,TB=2,CP=2,RC2=5,RC1=4;
%subplot(2,1,1)
for i=0
    for j=0.1:0.2:1
        for k=0.1:0.2:1
            for l=0.1:0.2:1
            [t,y]=ode45(@(t,y)
sifang(t,y,a,phi,b,Rr,Fr,Crh,Crl,Rm,Fm,psi,Cml,Cmh,Cgl,Cgh,Ng,Tg,Np,Tp,Cp),[0 50],[i j k l]);
            grid on
            plot3(y(:,1),y(:,2),y(:,4),'linewidth',1);
            set(gca,'XTick',[0:0.2:1],'YTick',[0:0.2:1],'ZTick',[0:0.2:1])
            set(gca,'XTickLabel',num2str(get(gca,'XTick')','%.1f'));
            set(gca,'YTickLabel',num2str(get(gca,'YTick')','%.1f'));
            set(gca,'ZTickLabel',num2str(get(gca,'ZTick')','%.1f'));
            hold on
            axis([0 1 0 1 0 1])
            end
        end
    end
end
xlabel('$r$','interpreter','latex');
ylabel('$m$','interpreter','latex');
zlabel('$p$','interpreter','latex','Rotation',360);
title('g=0','interpreter','latex');
%%%%%%%%%%%%%%%%%%%%%%%%%%%%%%
clc;clear;
PA=2,S1=9,L1CLG=3,S2=7,F=2,CS=1,PB=1,RI=6,RN=5,EC=0.5,SL=0.5,BB=0.5,E=1,L2CLT=3,RG
=8,RT=15,TB=2,CP=2,RC2=5,RC1=4;
%subplot(2,1,2)
for i=0. 8
    for j=0.1:0.2:1
        for k=0. 1:0.2:1
            for l=0.1:0.2:1
            [t,y]=ode45(@(t,y)
sifang(t,y,a,phi,b,Rr,Fr,Crh,Crl,Rm,Fm,psi,Cml,Cmh,Cgl,Cgh,Ng,Tg,Np,Tp,Cp),[0 50],[i j k l]);
            grid on
            plot3(y(:,1),y(:,2),y(:,4),'linewidth',1);
            set(gca,'XTick',[0:0.2:1],'YTick',[0:0.2:1],'ZTick',[0:0.2:1])
            set(gca,'XTickLabel',num2str(get(gca,'XTick')','%.1f'));
            set(gca,'YTickLabel',num2str(get(gca,'YTick')','%.1f'));
            set(gca,'ZTickLabel',num2str(get(gca,'ZTick')','%.1f'));
            hold on
            axis([0 1 0 1 0 1])
            end
        end
    end
end
xlabel('$r$','interpreter','latex');
ylabel('$m$','interpreter','latex');
zlabel('$p$','interpreter','latex','Rotation',360);
title('{\it\fontname{Bodoni MT}g}=1');
```

**The simulation tool used is Matlab2017a.The remaining simulation figures 7-11 were**

**obtained by modifying the relevant parameters based on the above program.**

**The specific parameter assignments are as follows:**

**Scenario 1:** According to the estimation of the actual situation, condition (1) and condition (2) are satisfied by the parameter assignment[27, 37]. The assignment of parameters in the evolutionary game system is as follows: $P_A = 2$ , $S_1 = 9$ , $\lambda_1 CL_G = 3$ , $S_2 = 7$ , $F = 2$ , $C_S = 1$ , $P_B = 1$ , $R_{ICEV} = 6$ , $R_{NEV} = 5$ , $\Delta_{EC} = 0.5$ , $\Delta_{SL} = 0.5$ , $\Delta_{BB} = 0.5$ , $\Delta_E = 1$ , $\lambda_2 CL_T = 3$ , $R_G = 8$ , $R_T = 15$ , $T_B = 2$ , $C_P = 2$ , $R_{C2} = 5$ , $R_{C1} = 4$ .

**Scenario 2:** According to the estimation of the actual situation, conditions (3), (4), and (5) are satisfied by parameter assignment. The assignment of parameters in the evolutionary game system is as follows: $P_A = 2$ , $S_1 = 9$ , $\lambda_1 CL_G = 3$ , $S_2 = 7$ , $F = 2$ , $C_S = 1$, $P_B = 1$, $R_{ICEV} = 16$ , $R_{NEV} = 5$ , $\Delta_{EC} = 0.5$ , $\Delta_{SL} = 0.5$ , $\Delta_{BB} = 0.5$ , $\Delta_E = 1$, $\lambda_2 CL_T = 3$, $R_G = 8$ , $R_T = 15$ , $T_B = 2$ , $C_P = 2$ , $R_{C2} = 5$ , $R_{C1} = 4$ .
